# Integrative Metabolomics and Machine Learning Approach to Uncover Scab Resistance Markers in Mature Pecan Trees

**DOI:** 10.1021/acs.jafc.5c16064

**Published:** 2026-04-29

**Authors:** Min Jeong Kang, Ronald B. Pegg, William L. Kerr, M. Lenny Wells, Patrick J. Conner, Joon Hyuk Suh

**Affiliations:** † Department of Food Science and Technology, College of Agricultural and Environmental Sciences, 1355 University of Georgia, 100 Cedar Street, Athens, Georgia 30602, United States; ‡ Department of Horticulture, College of Agricultural and Environmental Sciences, University of Georgia, 2360 Rainwater Road, Tifton, Georgia 31793, United States

**Keywords:** Mature pecan trees, Scab disease, Pathway-based metabolomics, Disease resistance, Biomarkers, Machine learning

## Abstract

Scab, caused by *Venturia effusa*, is the most destructive disease affecting pecan (*Carya illinoinensis*). Developing scab-resistant cultivars offers a sustainable solution for disease management. To elucidate the metabolic basis of resistance, we applied a pathway-based metabolomics that examines metabolites within pathways relevant to plant–pathogen interactions across 15 orchard-grown pecan cultivars with varying resistance levels. Machine learning models including support vector machine, random forest, and linear regression identified biomarkers predictive of resistance, achieving over 97% classification accuracy. Resistant cultivars showed elevated levels of metabolic precursors supporting lignin biosynthesis, energy metabolism, and antioxidant turnover, such as syringin, proline, and galactono-1,4-lactone, indicating a proactive and coordinated defense strategy. In contrast, susceptible trees accumulated defense-related end-products (e.g., salicylate glucoside, ascorbic acid) and nitrogen-rich amino acids, but showed limited availability of precursors, potentially contributing to increased susceptibility. These metabolic traits will support breeding of disease-resilient cultivars and improve disease management strategies in pecan orchards.

## Graphical abstract

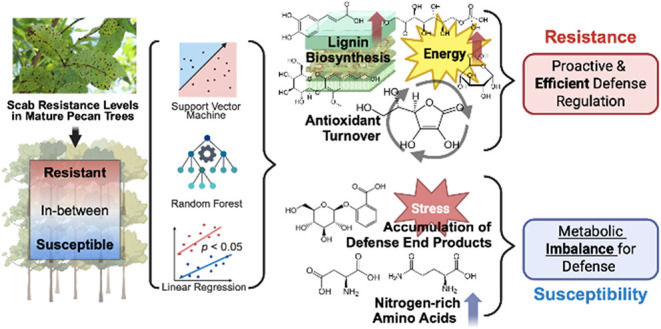

## 1 Introduction

Pecan (*Carya illinoinensis*), a tree nut native to North America, is an economically important crop in the United States. In 2023, the average national yield was 616 pounds per acre, with a total production value estimated at $460 million.^1^ Nearly 45% of commercial pecan production is concentrated in the Southeastern Coastal Plain, spanning the coastal regions of Virginia south to Georgia and west to Louisiana.^2^ While this region’s warm, humid climate supports optimal nut development, it also creates favorable conditions for pecan scab, the most destructive fungal disease affecting the crop in the southeastern U.S.^3^

Pecan scab, caused by *Venturia effusa* (*Fusicladium effusum*), infects leaves, twigs, and fruit shucks.^4^ Expanding leaves are particularly vulnerable, while developing fruits remain susceptible throughout maturation.^5^ During wet growing seasons, yield losses in susceptible cultivars can reach up to 100% if the disease is not adequately controlled.^6^ Current scab management relies heavily on fungicide applications, often repeated throughout the growing season.^7^ However, frequent rainfall can wash off treatments and accelerate disease spread, reducing fungicide efficacy.^8^ Furthermore, the emergence of fungicide-resistant *V. effusa* strains has diminished the long-term reliability of chemical control.^9^ Together, the escalating costs of fungicide use, declining effectiveness, and environmental concerns underscore the need for sustainable scab management strategies.^10^

One promising long-term strategy for scab management is the development of resistant pecan cultivars, which could reduce the need for chemical control.^11^ However, breeding for durable resistance remains challenging due to the presence of multiple pathogen races, each capable of infecting specific host genotypes.^3^ As pathogens adapt to host resistance, some cultivars previously considered resistant—such as the widely planted “Desirable”—have become increasingly susceptible in commercial orchards.^3^ Moreover, scab susceptibility often varies by region, likely due to geographic differences in pathogen race composition.^12^

Plants respond to fungal attack by modulating gene expression and activating defense-associated metabolic pathways.^13^ Metabolites serve as downstream indicators of these regulatory responses, capturing physiological and biochemical changes. This allows researchers to assess the final outcome of gene expression, enzyme activity, and environmental interactions.^14^ As such, metabolomics complements genomic and transcriptomic approaches, providing insights into the regulation and expression of resistance traits.^15–17^ While resistance conferred by specific genotypes may erode over time as pathogens evolve, the core metabolic responses to infection may be conserved across resistant cultivars. Identifying such shared metabolic features could advance our understanding of durable resistance mechanisms and guide breeding strategies.

Metabolomics has been applied to elucidate resistance mechanisms in diverse plant-fungal pathogen systems, including apple scab resistance,^17^ grapevine to fungal/oomycete-associated disease,^15^ and wheat resistance to Fusarium head blight (FHB).^18^ In pecan, a prior targeted comparison between the resistant “Kanza” and susceptible “Pawnee” cultivars identified quercetin derivatives as potential resistance biomarkers.^19^ However, this study was limited to a single cultivar pair, leaving open the question of whether common metabolic mechanisms are shared across a broader set of resistant genotypes. Complementing this, a transcriptomic analysis of ten field-grown pecan trees revealed that genes associated with leucine-rich repeat (LRR) receptors and mitogen-activated protein kinases (MAPKs)—key regulators of amino acid metabolism and hormone-mediated defense—were downregulated in resistant trees compared to susceptible ones.^20^ These findings emphasize the need for a broader, multigenotype metabolomic investigation under field conditions to bridge the gap between gene-level information and phenotypic expression, and to uncover robust metabolic signatures associated with scab resistance in pecan.

In this study, we hypothesize that scab-resistant mature pecan trees share conserved metabolic signatures that reflect underlying defense mechanisms. By identifying metabolites consistently associated with resistance across genetically diverse cultivars, we aim to uncover core biochemical pathways involved in durable scab resistance. In parallel, we also examine susceptible cultivars to identify biochemical signatures that may reflect vulnerabilities to infection. To test this, we conducted a pathway-based metabolomics analysis across 15 cultivars—classified as resistant, susceptible, or intermediate (in-between)—grown under identical orchard conditions for 15–20 years (mature trees). Samples were collected across two consecutive seasons, and machine learning techniques were applied to identify key metabolites and pathways predictive of resistance. Our findings provide insight into the metabolic basis of durable scab resistance and may inform future breeding strategies aimed at sustainable disease management in pecan.

## 2 Materials and Methods

### 2.1 Chemicals

Authentic chemical standards were obtained from various vendors to accurately identify the target metabolites. 2,5-Dihydroxybenzoic acid, abscisic acid, adenine, adenosine, adenosine 5′-diphosphate (ADP), alanine, arabinose, arabitol, arginine, ascorbic acid, asparagine, aspartate, benzoic acid, caffeic acid, catechin, *cis*-aconitate, citric acid, dehydroascorbate, deoxyadenosine, *trans*-ferulic acid, fructose, fumaric acid, galactono-1,4-lactone, gallic acid, gallocatechin, d-(+)­glucose, glutamic acid, glutamine, glycerate, guanosine, guanosine-5′-triphosphate (GTP), guanosine 5′-diphosphate-d-mannose (GDP-mannose), *trans*-4-hydroxy-l-proline, hypoxanthine, indole-3-acetic acid, indole-3-carboxylic acid, inosine, isoleucine, jasmonic acid, leucine, lysine, maleic acid, malic acid, malonic acid, methionine, *myo*-inositol, naringenin, ornithine, *p*-coumaric acid, phenylalanine, pinobanksin, l-proline, pyruvic acid, quercetin, quinic acid, d-(+)-raffinose, d-(−)-ribose, salicylic acid (SA), serine, sucrose, shikimic acid, sinapic acid, sissotrin (biochanin A-7-*O*-β-d-glucopyranoside), d-sorbitol, stachyose, succinic acid, tartaric acid, taxifolin (dihydroquercetin), *trans*-zeatin, *trans*-zeatin riboside, tryptophan, tyrosine, uric acid, valine, α-ketoglutaric acid, xylose, and γ-aminobutyric acid (GABA) were purchased from Sigma-Aldrich (St. Louis, MO, USA). 3-phosphoglycerate, adenosine 5′-monophosphate (AMP), adenosine 5′-triphosphate (ATP), apigenin 7-*O*-Neohesperidoside (rhoifolin), kaempferol-3Rh (kaempferol 3-*O*-α-L-rhamnoside), apigenin 7-*O*-glucoside (apigenin-7G), biochanin A (4’-methyl genistein), calycosin, chrysin, chrysoeriol, coniferyl alcohol, daidzein, dihydrokaempferol, dihydromyricetin, dihydroxyacetone phosphate (DHAP), dimethylallyl diphosphate (DMAPP), formononetin, galangin, genistein, genistein 7-*O*-d-glucoside (genistein-7G), d-glucuronate, d-glucosamine, glucose 6-phosphate (glucose-6P), glyceraldehyde 3-phosphate (GAD-3P), guanosine 5′-diphosphate (GDP), guanosine 5′-monophosphate (GMP), isoquercetin (quercetin 3-*O*-glucoside), jasmonic acid-isoleucine (JA-Ile), kaempferol 3-*O*-galactoside (trifolin), kaempferol 3-*O*-rutinoside (nictoflorin), luteolin 7-*O*-glucuronide, luteolin 7-*O*-glucoside, *N*-acetyl-d-glucosamine (GlcNAc), naringenin chalcone, naringin, phosphoenol pyruvic acid (PEP), pinocembrin, podophyllotoxin (PPT), 5-phospho-d-ribose 1-diphosphate (PRPP), prunetin, prunin (naringenin 7-*O*-glucoside), quercitrin (quercetin 3-*O*-rhamnoside), rutin (quercetin 3-*O*-rutinoside), baimaside (quercetin 3-*O*-sophoroside), sakuranetin, (−)­secoisolariciresinol, sedoheptulose 7-phosphate (sedoheptulose-7P), syringin, *N*,*N*′,*N*″-triacetylchitotriose, tricin, uridine diphosphate glucose (UDP-glucose), UDP-glucuronic acid, and β-glucogallin were purchased from Cayman Chemical (Ann Arbor, MI, USA). Apigenin, brassinolide, 5-caffeoylquinic acid (5-CQA), diacetylchitobiose, epigallocatechin gallate (EGCG), eriodictyol, kaempferol, luteolin, *p*-coumaraldehyde, *p*-coumaryl alcohol, salicylate 2-*O*-β-d-glucoside (SAG), tricetin, and vestitone were purchased from Toronto Research Chemicals Inc. (North York, ON, Canada). Citrulline, 5-aminopentanoate, acetyl-l-lysine, isocitric acid, glycolate, oxalic acid, salicin, threonic acid, *trans*-cinnamic acid, and trehalose were obtained from Fisher Scientific (Fair Lawn, NJ, USA). For internal standards, genistein-*d*_4_, l-proline-2,5,5-*d*_3_, salicylic acid-*d*_4_, and phenylalanine-^13^C_6_ were purchased from Cayman Chemical (Ann Arbor, MI, USA) and hippuric acid-*d*_5_ was obtained from Toronto Research Chemicals Inc. (North York, ON, Canada). *N*,*N*-dimethyl-*d*_6_-glycine and methionine-*d*_3_ were purchased from CDN Isotopes Inc. (Pointe-Claire, QC, Canada). Acetonitrile, water, and isopropyl alcohol were of LC-MS grade and were obtained from Fisher Scientific (Fair Lawn, NJ, USA). Formic acid (>99%) was LC-MS grade and purchased from Sigma-Aldrich (St. Louis, MO, USA). All chemicals and reagents were of analytical grade.

### 2.2 Plant Materials

Pecan cultivars with varying levels of resistance to pecan scab were selected based on multiyear assessments of disease severity, in consultation with pecan breeders and horticulturists at the University of Georgia.^11,21–23^ Fifteen cultivars were selected and were categorized into three resistance groups; (1) Resistant: “Avalon”, “Elliott”, “Lakota”, “Excel”, and “McMillan”/ (2) Intermediate (In-between): “Cape Fear”, “Sumner”, “Oconee”, “Creek”, and “Kalos”/ (3) Susceptible: “Desirable”, “Pawnee”, “Morrill”, “Byrd”, and “Treadwell” (Figure 1). All cultivars were grown at the University of Georgia’s Ponder Research Farm (31°30′34.88″N, 83°38′55.26″W). Trees in the orchard were 15–20 years old and spaced within a 500 m radius, with consistent irrigation and fertilization. Leaf samples were collected over two consecutive years on September 13, 2023, and September 9, 2024. For each cultivar, five biological replicates (*n* = 5) were sampled, except for “Treadwell” (*n* = 4), which was affected by hurricane damage (Helene, 2024). In both sampling years, the same total number of leaf samples was collected, yielding 74 samples per year. Although the ideal experimental design includes both healthy and diseased individuals across genotypes, practical field constraints, e.g., low disease incidence during the sampling seasons, limited our ability to collect diseased samples. Therefore, only healthy, fully expanded, and mature leaflets (i.e., hardened-off and free from visible pathogen or insect damage) were collected and analyzed. Samples were harvested from the sun-exposed exterior canopy, ensuring at least 50% daily sunlight exposure. From each tree, five leaves were randomly selected, and from each leaf, the third pair of leaflets (counting from the tip) was excised, yielding 10 leaflets per tree. Samples were flash-frozen in liquid nitrogen immediately upon collection, stored on dry ice, and later transferred to a −80 °C freezer until further analysis.

**Figure 1:**
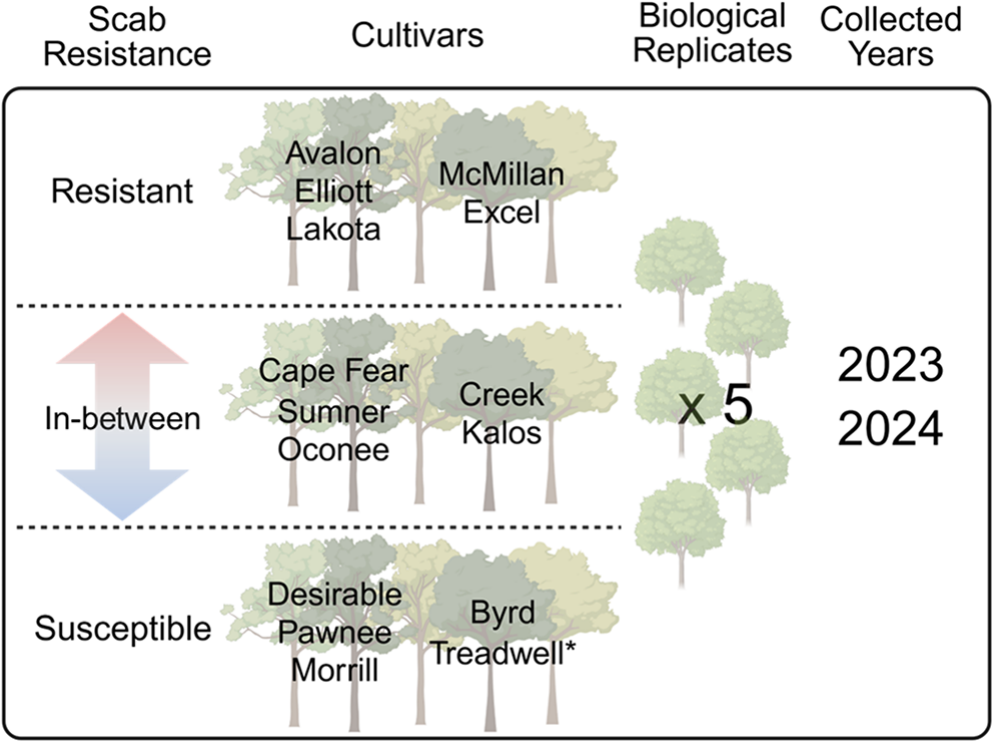
Overview of the experimental design.

### 2.3 Sample Preparation

Frozen leaf samples were lyophilized for 48 h and then finely ground under liquid nitrogen using a mortar and pestle. For reverse-phase (RP) chromatography, 35 mg of dry powder was extracted with 1.0 mL of chilled acetonitrile/isopropanol/water (3:3:2, v/v/v) containing internal standards, including hippuric acid-*d*_5,_ genistein-*d*_4_, apigenin-*d*_5_, and salicylic acid-*d*_4._ For hydrophilic interaction chromatography (HILIC), 35 mg of powder was extracted with 1.0 mL of 20% methanol containing internal standards, including alanine-*d*_3_, *N*,*N*-dimethyl-*d*_6_-glycine, methionine-*d*_3,_ phenylalanine-^13^C_6,_ and l-proline-2,5,5-*d*_3_. Extraction was performed by vortexing for 10 min followed by 30 min of sonication in an ice-cooled ultrasonic bath. Samples were centrifuged at 21,000*g* for 10 min at 4 °C, and the resulting supernatant was collected. An aliquot of 200 μL was filtered through a 0.22-μm PVDF membrane filter (Cytiva, Marlborough, MA, USA) and was injected into the liquid chromatography–mass spectrometry (LC–MS) system for metabolite profiling. Quality control (QC) samples were prepared by pooling equal volumes (5 μL) of each extract and were injected at regular intervals throughout the analytical run (within batch and batch-to-batch) to monitor instrument stability and batch effects. The mixtures of internal standards were also analyzed at the beginning, middle, and end of each batch as an external QC. All sample injections were randomized to minimize systematic biases.

### 2.4 Pathway-Based Metabolomics

Metabolomic analysis was performed using an Agilent 1260 Infinity II UHPLC coupled with a 6470 Triple Quadrupole (QqQ) mass spectrometer (Agilent Technologies, Santa Clara, CA, USA). Pathway-based metabolomics is a targeted approach in which analytes are selected a priori based on their roles in specific biological pathways relevant to the research question, rather than being profiled in an untargeted manner. Targeted metabolites used in this study were selected based on their known involvement in primary and secondary metabolic pathways relevant to plant-pathogen interactions (Supporting Table S1). Chromatographic separation was conducted using RP and HILIC under different conditions with distinct mobile phases.

For RP chromatography, an Acclaim C30 column (2.1 mm × 150 mm, 3 μm, Thermo Scientific, Waltham, MA, USA) was utilized to mainly analyze organic acid, flavonoids, and plant hormones. The mobile phase comprised water with 0.1% formic acid (A) and acetonitrile with 0.1% formic acid (B). Gradient conditions were: 2% B (0–2.5 min), 20–50% B (2.6–19 min), 50–90% B (19–25 min), 95% B (25.1–35 min), and a re-equilibration period at 2% B for another 10 min. Flow rate and column temperature were maintained at 0.2 mL/min and 30 °C, respectively.

For the HILIC based separation, sugars, amino acids, nucleotides, and organic acids were primarily analyzed using a Poroshell 120 HILIC-Z column (2.1 mm × 150 mm, 2.7 μm, Agilent Technologies, Santa Clara, CA, USA). The mobile phase consisted of 10 mM ammonium acetate (pH 9.0) in water (A) and in 90% acetonitrile (B) with 5 μM InfinityLab Deactivator Additive (Agilent Technologies, Santa Clara, CA, USA). The gradient elution was as follows: 90% B (0–2 min), 90–70% B (2–6 min), 70–40% B (6–7.5 min), 40% (7.5–15 min), followed by re-equilibration with 90% B (9 min). The flow rate was 0.25 mL/min, and column temperature was maintained at 30 °C.

The MS detection utilized electrospray ionization (ESI) in both positive and negative ionization modes. Dynamic multiple reaction monitoring (DMRM) was applied, with MS/MS parameters optimized via standard injections (Supporting Table S1). ESI settings included: Gas temperature 275 °C; gas flow 11 L/min; nebulizer pressure 45 psi; sheath gas temperature 325 °C; sheath gas flow 11 L/min; capillary spray voltage +3.5 kV (for positive mode) and −2.5 kV (for negative mode).

### 2.5 Data Processing and Statistical Analysis

Metabolomics data from LC–MS analyses were processed using MassHunter Quantitative Analysis 10.1 software (Agilent Technologies, Santa Clara, CA, USA). Relative metabolite abundance was quantified by comparing analyte peak areas against internal standards. Quality control (QC) criteria required the relative standard deviation (RSD) of QC samples to be ≤30%. The variation in metabolic profiles was evaluated using principal component analysis (PCA) and partial least-squares discriminant analysis (PLS-DA), performed in R (https://www.r-project.org). Figures were generated using R and BioRender.

The workflow to identify biomarkers distinguishing scab-resistant from scab-susceptible cultivars was summarized in Supporting Figure S1, and support vector machine (SVM) and random forest (RF) classification algorithms were applied. SVM classification was conducted using the e1071 package with a linear kernel and a cost parameter of 1, selected based on internal cross-validation performance. RF models were implemented using the randomForest package with ntree = 500 and mtry = floor­(sqrt­(*p*)), where *p* is the number of predictors. Hyperparameters for both models were chosen through repeated cross-validation within the training set to reduce overfitting and ensure reproducibility. For binary classification, resistance was coded as “1” and susceptibility as “0”, enabling interpretation of SVM coefficients in terms of directionality. After normalization, model performance was evaluated using 10-fold cross-validation, where 30% of the data was randomly assigned to a test set, and the remaining 70% served as the training set. This procedure was repeated 10 times with nonoverlapping subsets. Average classification performance was computed across all iterations. Performance metrics, derived from the confusion matrix, included:

- True Positive (TP)–the number of resistant trees correctly classified.
- False Positive (FP)–the number of susceptible trees misclassified as resistant.
- True Negative (TN)–the number of susceptible trees correctly classified.
- False Negative (FN)–the number of resistant trees misclassified as susceptible.

From these, accuracy, specificity, sensitivity, precision, F1-score, and Matthews correlation coefficient (MCC) were calculated.^24^

To control year-to-year environmental variation, biomarker selection was conducted independently for each year (2023 and 2024). Only metabolites showing consistent direction of association (based on SVM coefficients) across both years were retained. Biomarkers exhibiting conflicting directionality between years were excluded. This cross-year consistency filter reduces the likelihood of selecting features driven by year-specific environmental variation, thereby prioritizing metabolites that reflect stable resistance-associated metabolic differences. Although this year-by-year approach will reduce the sample size available for model training in each subset, the consistency of selected biomarkers across both years supports the robustness of the identified metabolites. Subsequently, linear regression models were fitted using R to verify the significance and directionality of group differences. Positive SVM coefficients and linear model estimates indicated association with resistance, while negative values indicated association with susceptibility. Permutation testing (1,000 iterations with randomly shuffled class labels) was performed to evaluate whether classification performance exceeded chance levels. *P*-values were adjusted for multiple comparisons using the false discovery rate (FDR) method. Metabolites with FDR-adjusted *p*-values <0.05 were considered statistically significant. All machine learning approaches were performed in R (https://www.r-project.org).

#### 2.5.1 Effect Size Analysis (Cohen’s *d*)

Prior to effect size calculation, metabolite intensities were log_2_ transformed using a pseudocount of 1 (i.e., log_2_(*x* + 1)) to stabilize variance and reduce right-skewness in the raw LC–MS/MS peak intensities. Cohen’s *d* was then used to quantify standardized differences in log_2_ scaled metabolite abundance across different resistance levels.^25^

For susceptibility biomarkers, the resistant group was used as the reference because these metabolites are expected to be least abundant in highly resistant cultivars. For resistance biomarkers, the susceptible group served as the reference because these metabolites are expected to be least abundant in highly susceptible cultivars. For each metabolite and comparison group *g*, Cohen’s *d* was computed from replicate-level log_2_ intensities as

$$d_{g}=\frac{{\mathrm{X̅}}_{g}-{\mathrm{X̅}}_{\mathrm{ref}}}{s_{p}}$$

where *X̅_g_* and *X̅_ref_* are the mean log_2_ transformed abundances of group *g* and the reference group (*ref*), respectively. The pooled standard deviation was calculated using the classical two sample pooled variance estimator

$$s_{p}=\sqrt{\frac{\left(n_{g}-1\right)s_{g}^{2}+\left(n_{\mathrm{ref}}-1\right)s_{\mathrm{ref}}^{2}}{n_{g}+n_{\mathrm{ref}}-2}}$$

where *n* and *s* denote the number of biological replicates and their sample standard deviation. This formulation expresses differences between groups in standardized units of shared variability.^25^

Cohen’s *d* values were visualized as heatmaps without row scaling to preserve effect-size magnitude. A five-color palette (“darkblue,” “steelblue,” “white,” “pink,” and “firebrick”) was applied to a symmetric range of −1.5 to +1.5, corresponding to Cohen’s *d* values of −1.5, −0.5, 0, +0.5, and +1.5, respectively. According to Cohen’s guidelines, effect sizes of approximately 0.2–0.3 are considered small, values around 0.5 represent medium effects, and values ≥0.8 indicate large effects.^26^ Thus, any nonwhite color in the heatmap corresponds to at least a medium effect size. Positive values indicate higher metabolite abundance relative to the reference group, whereas negative values indicate lower abundance.

## 3 Results and Discussion

### 3.1 Metabolic Variation among Pecan Cultivars with Varying Levels of Scab Resistance

The metabolite profiles of 15 pecan cultivars were identified and characterized using a pathway-based metabolomics approach, with a primary focus on biological pathways associated with plant–pathogen interactions.^27–30^ Relevant primary metabolic pathways, including the tricarboxylic acid (TCA) cycle, urea cycle, ascorbic acid biosynthesis, amino acid metabolism, and carbohydrate metabolism, and secondary metabolic pathways, including flavonoid biosynthesis, plant hormone regulation, phenylpropanoid, and lignin biosynthesis, were investigated. In total, 171 metabolites were targeted within the pathways, comprising 12 sugars, 34 organic acids, 19 amino acids, 10 plant hormones, 50 flavonoids, and 46 metabolites from other classes (Supporting Table S1). The identities of the majority of these metabolites were confirmed using authentic chemical standards. Analytical results from internal and external QC samples demonstrated both within-batch and batch-to-batch reproducibility, with RSDs below 20% in most cases, except for a few outliers (general criterion for metabolomics: RSD < 30%) (Supporting Table S2).

To evaluate overall metabolic differences among the cultivars, PLS-DA was employed. When all groups (resistant, intermediate, and susceptible) were included, the PLS-DA model did not exhibit strong classification performance (*Q* ^2^ < 0.5; data not shown), suggesting limited discriminatory power, likely due to the overlapping metabolic features of intermediate cultivars (in-between group). However, a valid and robust model was obtained when comparing the resistant and susceptible groups (*R* ^2^ *Y* = 0.88, *Q* ^2^ = 0.59, RMSE_cv_ = 0.19) with statistical significance confirmed by a permutation test (*pR* ^2^ *Y* = 0.007, *p*Q^2^ = 0.001) (Figure 2). These results indicate that the metabolic profiles of scab-resistant and -susceptible cultivars are distinctly different.

**Figure 2:**
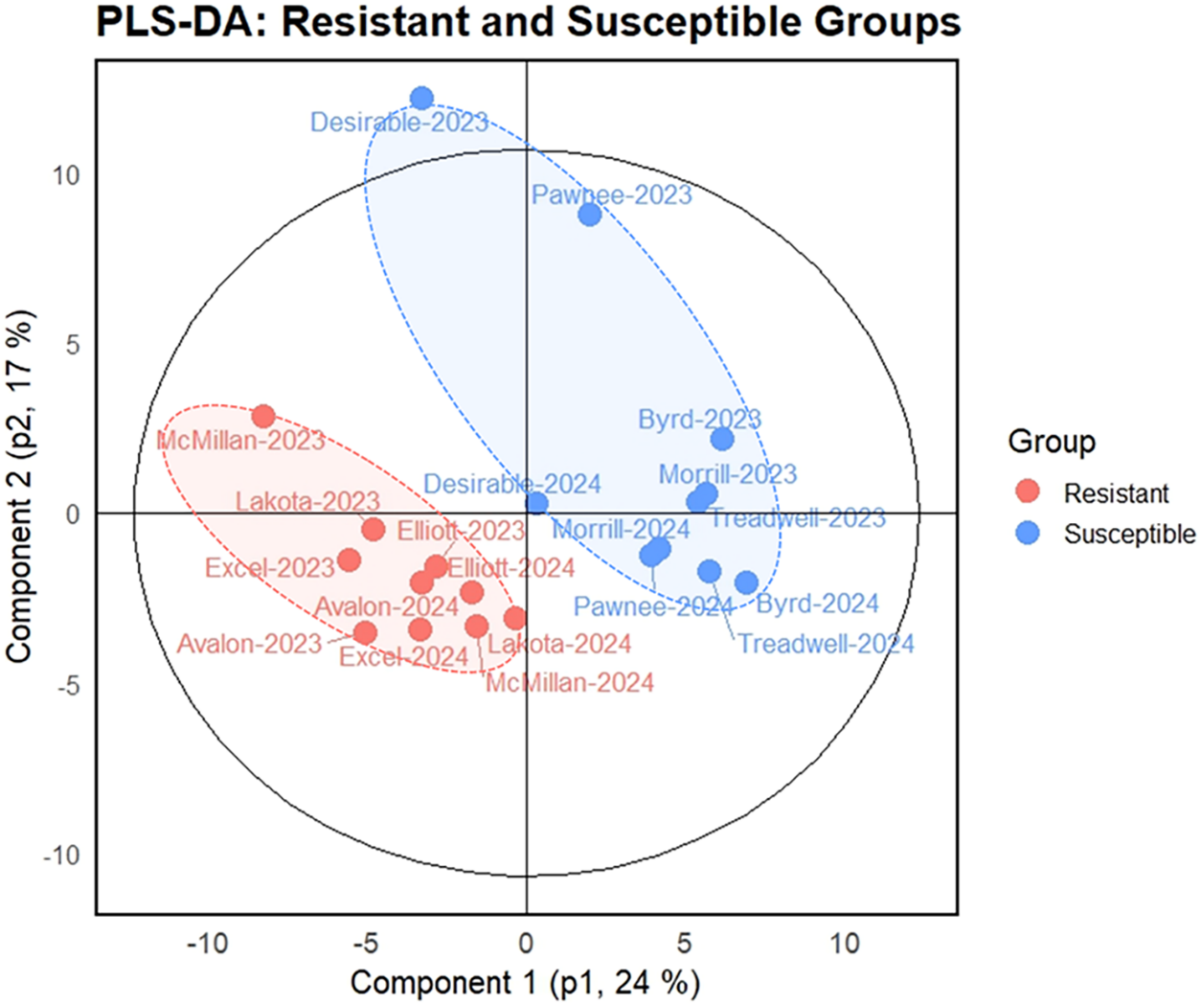
Partial least-squares discriminant analysis (PLS-DA) of metabolite profiles in scab-resistant and susceptible pecan cultivars across two consecutive years. The model showed valid performance (*R* ^2^ *Y* = 0.88, *Q* ^2^ = 0.59, RMSE_cv_ = 0.19), with statistical significance confirmed by permutation testing (*pR* ^2^ *Y* = 0.007, *pQ* ^2^ = 0.001).

Nonetheless, PLS-DA carries an inherent risk of overfitting when applied to high-dimensional data sets.^31^ Indeed, a previous study reported overfitting issues with PLS models in metabolomics data sets due to dimensionality reduction.^32^ Therefore, although PLS-DA provided evidence of group separation, it was not considered sufficient for reliable biomarker discovery. To address this limitation and identify robust metabolites linked to resistance or susceptibility, machine learning algorithms were subsequently employed.

### 3.2 Machine Learning-Based Identification of Biomarkers Distinguishing Scab-Resistant and Susceptible Pecan Trees

Machine learning (ML) offers significant advantages for biomarker selection in metabolomics due to its capacity to handle complex, high-dimensional data sets.^33^ For binary classification tasks, support vector machine (SVM) has proven effective in identifying the optimal hyperplane that separates classes while minimizing classification error. Additionally, SVM provides coefficient values that reflect the contribution of each metabolite to class separation.^34,35^ Random forest (RF), an ensemble-based method, constructs multiple decision trees and aggregates their predictions to improve classification accuracy and robustness. RF can estimate feature importance by quantifying each metabolite’s contribution to correct classifications across the forest of trees.^36^ Given their complementary strengths, both SVM and RF were employed in this study to select robust biomarkers. Metabolites consistently identified by both models were considered meaningful markers associated with scab resistance or susceptibility.

Classification was performed to distinguish resistant and susceptible cultivars. The data set was first divided by separation method (RP and HILIC) and then stratified by collection year (2023 and 2024), as plant metabolomes are known to be influenced by environmental variation across seasons.^37^ Both SVM and RF were applied to each data set, and the resulting model performance metrics are summarized in Table 1. SVM achieved mean classification accuracies ranging from 98.00% to 100.00%, while RF accuracies ranged from 90.00% to 99.00%. These results confirmed the effectiveness of both models in discriminating scab resistance based on metabolite profiles. Permutation testing confirmed that observed classification accuracies significantly exceeded null distributions for all comparisons (*p* < 0.01; Supporting Figure S2).

**Table 1:** Average of Confusion Matrices and Performance Indicators from 10-Fold Cross-Validation for Classification between Resistant and Susceptible Groups by Support Vector Machine and Random Forest

Support Vector Machine											
		Confusion matrix^a^									
Mode	Year	TP	FP	FN	TN	Accuracy (%)	Sensitivity (%)	Specificity (%)	Precision (%)	F1 Score (%)	Matthews correlation coefficient (%)
RP	2023	5.80	0.00	0.10	4.10	99.00 ± 3.16	98.57 ± 4.52	100.00 ± 0.00	100.00 ± 0.00	99.23 ± 2.43	98.02 ± 6.27
	2024	5.10	0.00	0.20	4.70	98.00 ± 4.22	97.50 ± 5.27	100.00 ± 0.00	100.00 ± 0.00	98.67 ± 2.81	95.28 ± 9.96
HILIC	2023	4.30	0.00	0.00	5.70	100.00 ± 0.00	100.00 ± 0.00	100.00 ± 0.00	100.00 ± 0.00	100.00 ± 0.00	100.00 ± 0.00
	2024	5.20	0.00	0.20	4.60	98.00 ± 4.22	95.83 ± 9.00	100.00 ± 0.00	100.00 ± 0.00	97.66 ± 5.08	96.18 ± 8.05

Random Forest											
		Confusion matrix									
Mode	Year	TP	FP	FN	TN	Accuracy (%)	Sensitivity (%)	Specificity (%)	Precision (%)	F1 Score (%)	Matthews correlation coefficient (%)
RP	2023	5.20	0.20	0.00	4.60	98.00 ± 6.32	100.00 ± 0.00	96.00 ± 12.65	97.14 ± 9.04	98.33 ± 5.27	96.55 ± 10.92
	2024	5.70	0.70	0.30	3.30	90.00 ± 6.67	95.89 ± 9.45	80.74 ± 21.57	89.68 ± 9.71	91.98 ± 5.47	78.75 ± 14.47
HILIC	2023	5.20	0.10	0.00	4.70	99.00 ± 3.16	100.00 ± 0.00	96.67 ± 10.54	98.75 ± 3.95	99.33 ± 2.11	97.64 ± 7.47
	2024	5.20	0.20	0.50	4.10	93.00 ± 6.75	91.14 ± 13.43	94.67 ± 11.67	97.08 ± 6.23	93.27 ± 7.76	86.55 ± 12.48

a TP: true positive; FP: false positive; FN: false negative; TN: true negative.

To ensure the robustness of selected biomarkers across years, metabolites with inconsistent SVM coefficient directions between years (i.e., SVM coefficient switching from positive to negative between years or vice versa) were excluded, as such reversals suggest unstable or context-dependent associations. For the remaining metabolites, the mean values of SVM coefficients and RF importance scores across years were used to evaluate their relevance. Statistical validation was conducted using linear regression, with false discovery rate (FDR)-adjusted *p*-values to assess significance and direction of association. Metabolites with adjusted *p*-value ≥ 0.05 were excluded. The final list of biomarkers—those consistently selected by both SVM and RF, exhibiting stable directionality, and statistically significant differences between groups—is presented in Supporting Table S3 and visualized in Figure 3. These features displayed consistent SVM coefficient signs and regression estimates aligned with either resistance or susceptibility, supporting their reliability as candidate biomarkers.

**Figure 3:**
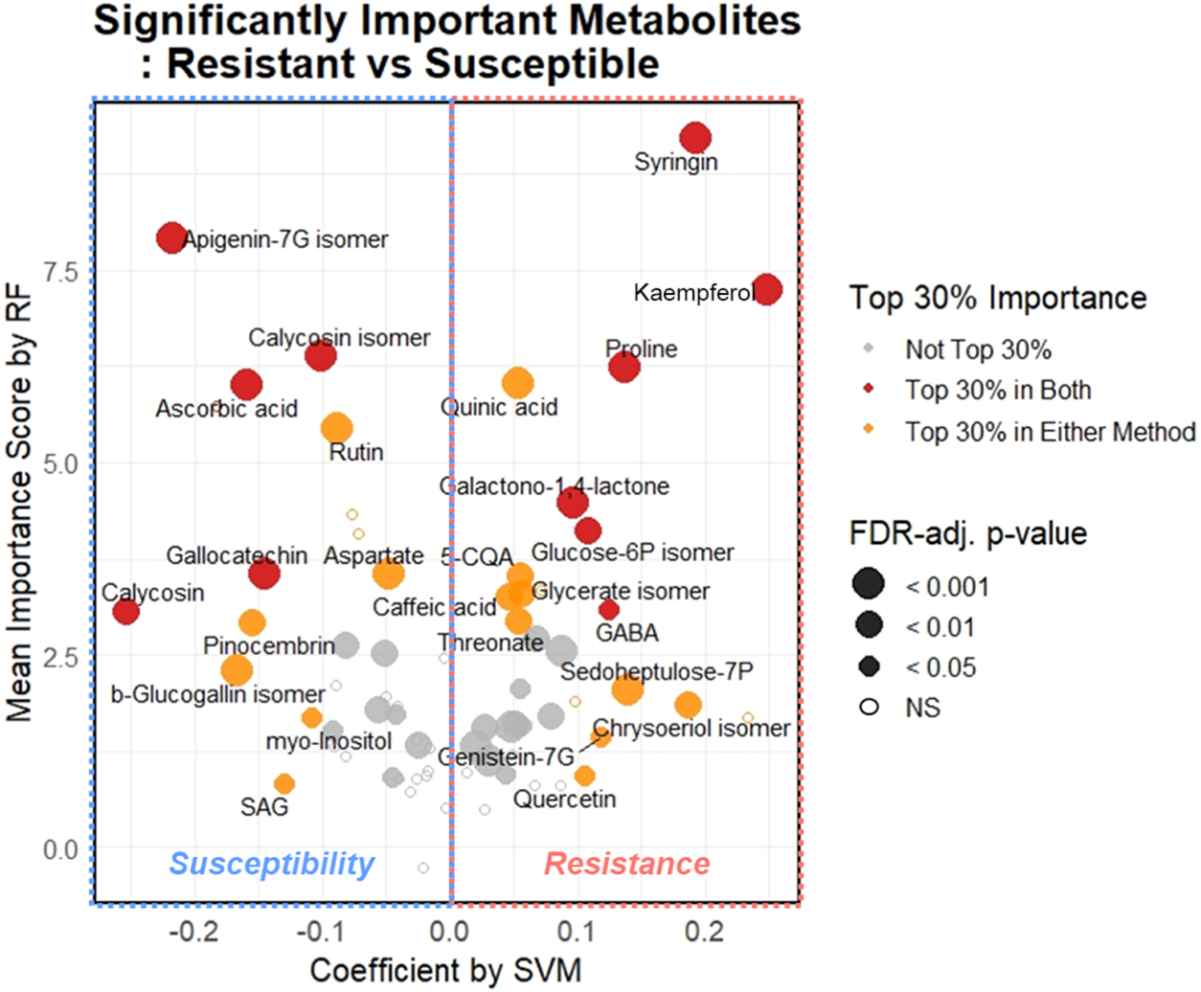
Significantly important metabolites identified by machine learning algorithms distinguishing resistant and susceptible cultivars. On the *x*-axis, positive SVM coefficients indicate association with the resistance group; negative coefficients indicate association with the susceptible group. On the *y*-axis, RF importance scores reflect the decrease in classification accuracy when a given metabolite is excluded; higher values denote greater contribution to model performance. Dot color represents whether the metabolite ranked in the top 30% of importance in either or both methods. Dot size reflects statistical significance based on linear regression; larger dots indicate lower FDR-adjusted *p*-values. Metabolites with no significant difference between groups are represented by hollow circles (NS, not significant, *p* ≥ 0.05).

Figure 3 presents 26 significant biomarkers, all of which ranked within the top 30% in importance by either or both ML methods. Fifteen metabolites were selected for resistance biomarkers, including syringin, kaempferol-3Rh, quinic acid, galactono-1,4-lactone, proline, threonate, glycerate isomer, sedoheptulose-7P, glucose-6P isomer, chrysoeriol isomer, GABA, quercetin, 5-CQA, caffeic acid, and genistein-7G. Eleven metabolites were identified as susceptibility biomarkers, including apigenin-7G isomer, calycosin isomer, rutin, ascorbic acid, aspartate, gallocatechin, β-glucogallin isomer, calycosin, SAG, pinocembrin, and *myo*-inositol. Among the resistance biomarkers, six compounds—syringin, kaempferol-3Rh, galactono-1,4-lactone, proline, glucose-6P isomer, and GABA—were consistently ranked as highly significant by both SVM and RF models. Likewise, five susceptibility biomarkers—apigenin-7G isomer, ascorbic acid, gallocatechin, calycosin, and calycosin isomer—exhibited high importance in both models. The biological relevance of these biomarkers, particularly their roles in plant defense–related metabolic pathways, will be discussed in detail in the following section on metabolic pathway mapping.

### 3.3 Discrimination of Pecan Cultivars with Intermediate Resistance Using Selected Biomarkers

To evaluate whether the biomarkers identified from the resistant and susceptible cultivars could also distinguish those with intermediate resistance (in-between group), PCA was conducted using the 26 selected biomarkers (Figure 4). “Oconee” cultivar was removed from in-between group due to its outlier profile. In this reduced space, intermediate cultivars separated into two distinct subgroups—“resistant-like” and “susceptible-like”—based on their resistance levels, as explained by 34.8% (PC1) and 10.7% (PC2) of the total variance. “Sumner” (2023 and 2024) and “Kalos” (2023 and 2024) were positioned closer to the resistant group, whereas “Cape Fear” and “Creek” (both years) aligned more closely with the susceptible group. These projections are consistent with previously reported scab resistance observations, where “Sumner” exhibited greater resistance compared to “Cape Fear”.^38^ These findings suggest that the selected biomarkers may also be effective for characterizing cultivars with intermediate resistance according to their relative degree of resistance.

**Figure 4:**
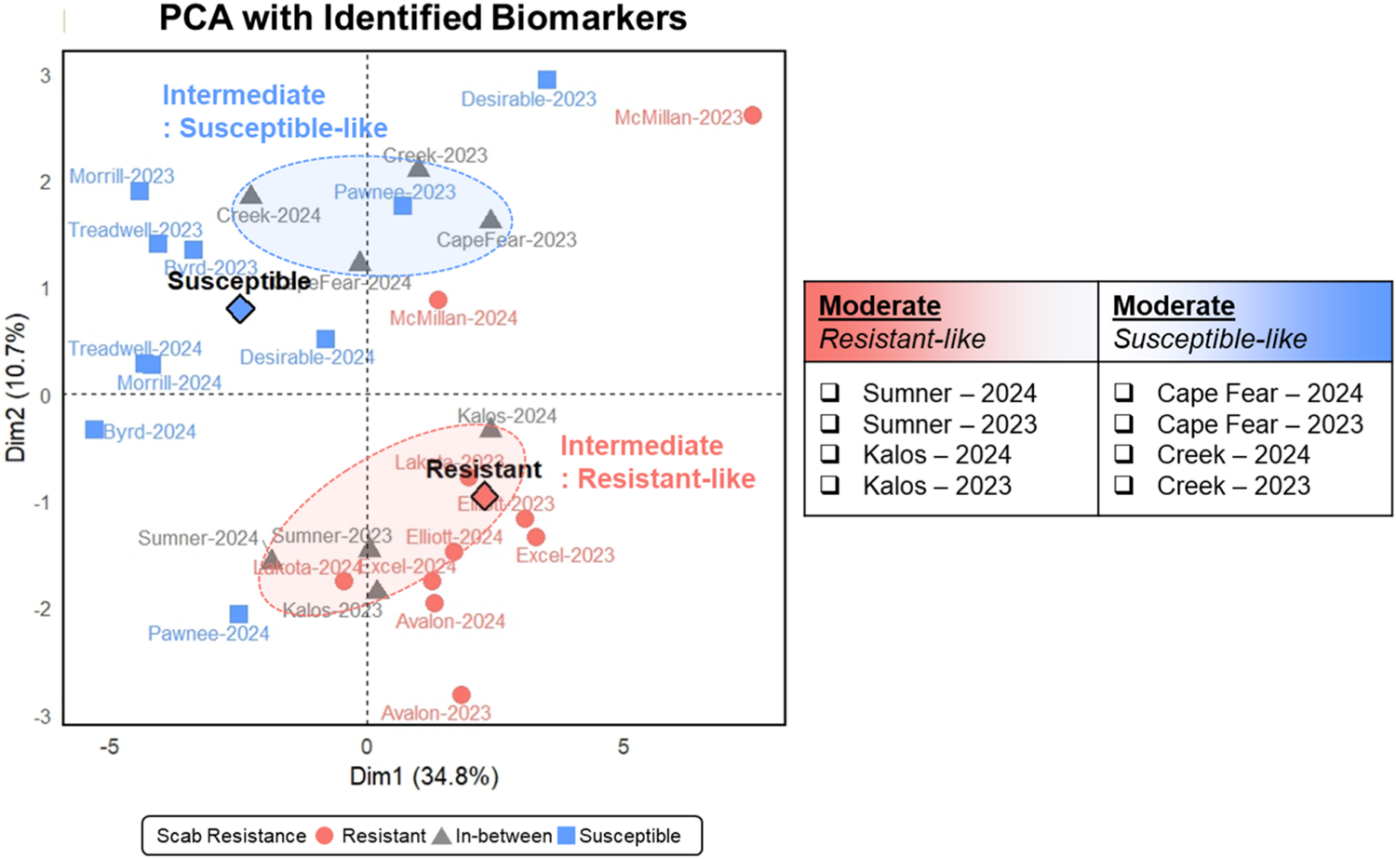
Discrimination of intermediate-resistance cultivars into resistant-like and susceptible-like subgroups using 26 metabolite biomarkers identified from the comparison between resistant and susceptible cultivars.

### 3.4 Extended Biomarker Discovery Involving Resistant-like and Susceptible-like Cultivars

Additional classification analyses were performed using resistant-like and susceptible-like cultivars to expand and validate the resistance- and susceptibility-associated biomarkers. Comparisons between “resistant and susceptible-like” cultivars aimed to strengthen the identification of resistance-associated biomarkers, while comparisons between “resistant-like and susceptible” cultivars supported the identification of susceptibility-associated biomarkers. This strategy enabled the evaluation of previously identified markers for consistency and the discovery of additional potential markers. The same ML algorithms (SVM and RF) were applied, and their performance is summarized in Table 2. Classification accuracy was high across all comparisons: for the resistant vs susceptible-like group, SVM achieved mean accuracies between 96.67% and 100.00%, and RF ranged from 91.11% to 100.00%. For the resistant-like vs susceptible group comparison, SVM accuracies ranged from 95.56% to 100.00%, and RF from 94.44% to 100.00%. These results confirm that both models reliably distinguish intermediate subgroups based on their metabolite profiles.

**Table 2:** Average of Confusion Matrices and Performance Indicators from 10-Fold Cross-Validation for Classification between Resistant vs Susceptible-like and Resistant-like vs Susceptible Groups by Support Vector Machine and Random Forest

Resistant vs Intermediate (Susceptible-like): Support Vector Machine											
		Confusion matrix^a^									
Mode	Year	TP	FP	FN	TN	Accuracy (%)	Sensitivity (%)	Specificity (%)	Precision (%)	F1 Score (%)	Matthews correlation coefficient (%)
RP	2023	6.00	0.00	0.30	2.70	96.67 ± 5.37	95.24 ± 7.69	100.00 ± 0.00	100.00 ± 0.00	97.41 ± 4.18	93.37 ± 10.72
	2024	6.40	0.00	0.10	2.50	98.89 ± 3.51	98.57 ± 4.52	100.00 ± 0.00	100.00 ± 0.00	99.23 ± 2.43	97.56 ± 7.72
HILIC	2023	6.60	0.00	0.00	2.40	100.00 ± 0.00	100.00 ± 0.00	100.00 ± 0.00	100.00 ± 0.00	100.00 ± 0.00	100.00 ± 0.00
	2024	6.40	0.00	0.20	2.40	97.78 ± 4.68	96.90 ± 6.55	100.00 ± 0.00	100.00 ± 0.00	98.32 ± 3.55	95.46 ± 9.60

Resistant vs Intermediate (Susceptible-like): Random Forest											
		Confusion matrix									
Mode	Year	TP	FP	FN	TN	Accuracy (%)	Sensitivity (%)	Specificity (%)	Precision (%)	F1 Score (%)	Matthews correlation coefficient (%)
RP	2023	6.30	0.60	0.20	1.90	91.11 ± 13.66	96.57 ± 7.35	80.00 ± 34.96	93.06 ± 14.16	93.93 ± 8.94	77.05 ± 7.71
	2024	6.40	0.10	0.10	2.40	97.78 ± 4.68	98.75 ± 3.95	97.50 ± 7.91	98.33 ± 5.27	98.42 ± 3.37	94.52 ± 11.95
HILIC	2023	6.30	0.40	0.00	2.30	95.56 ± 7.77	100.00 ± 0.00	88.33 ± 19.33	94.29 ± 9.99	96.79 ± 5.71	91.09 ± 14.97
	2024	6.70	0.00	0.00	2.30	100.00 ± 0.00	100.00 ± 0.00	100.00 ± 0.00	100.00 ± 0.00	100.00 ± 0.00	100.00 ± 0.00

Intermediate (Resistant-like) vs Susceptible: Support Vector Machine											
		Confusion matrixa									
Mode	Year	TP	FP	FN	TN	Accuracy (%)	Sensitivity (%)	Specificity (%)	Precision (%)	F1 Score (%)	Matthews correlation coefficient (%)
RP	2023	3.10	0.00	0.00	5.90	100.00 ± 0.00	100.00 ± 0.00	100.00 ± 0.00	100.00 ± 0.00	100.00 ± 0.00	100.00 ± 0.00
	2024	2.50	0.00	0.10	6.40	98.89 ± 3.51	96.67 ± 10.54	100.00 ± 0.00	100.00 ± 0.00	98.00 ± 6.32	97.56 ± 7.72
HILIC	2023	2.70	0.00	0.00	6.30	100.00 ± 0.00	100.00 ± 0.00	100.00 ± 0.00	100.00 ± 0.00	100.00 ± 0.00	100.00 ± 0.00
	2024	2.10	0.00	0.40	6.50	95.56 ± 5.74	84.17 ± 21.68	100.00 ± 0.00	100.00 ± 0.00	89.90 ± 14.17	88.69 ± 15.09

Intermediate (Resistant-like) vs Susceptible: Random Forest											
		Confusion matrix									
Mode	Year	TP	FP	FN	TN	Accuracy (%)	Sensitivity (%)	Specificity (%)	Precision (%)	F1 Score (%)	Matthews correlation coefficient (%)
RP	2023	2.30	0.00	0.00	6.70	100.00 ± 0.00	100.00 ± 0.00	100.00 ± 0.00	100.00 ± 0.00	100.00 ± 0.00	100.00 ± 0.00
	2024	1.90	0.10	0.40	6.60	94.44 ± 7.86	87.67 ± 20.25	98.75 ± 3.95	95.00 ± 15.81	88.83 ± 14.91	87.11 ± 16.93
HILIC	2023	1.80	0.00	0.30	6.90	96.67 ± 5.37	89.17 ± 18.45	100.00 ± 0.00	100.00 ± 0.00	93.24 ± 11.82	92.08 ± 13.14
	2024	3.00	0.40	0.10	5.50	94.44 ± 5.86	96.67 ± 10.54	92.57 ± 9.73	90.67 ± 12.65	92.67 ± 8.38	89.12 ± 11.58

a TP: true positive; FP: false positive; FN: false negative; TN: true negative.

To ensure the robustness of marker selection, metabolites with inconsistent SVM coefficient directions between years were excluded. Additionally, metabolites with opposite coefficient directions compared to the original resistant vs susceptible comparison (Section 3.2) were also removed to avoid contradictory signals (Supporting Table S4). Only metabolites with statistically significant differences (FDR-adjusted *p* < 0.05) and consistent directional association were retained. Top-ranking features from each comparison are shown in Figure 5A (resistance biomarkers) and Figure 5B (susceptibility biomarkers). Nine metabolites were identified as resistance biomarkers, such as GDP-mannose, glucose, serine, tyrosine, sedoheptulose-7P, sucrose, proline, glycerate isomer, and 5-CQA. Among these, four compounds—proline, glycerate isomer, 5-CQA, and quercetin—overlapped with those found in the original resistant vs susceptible classification (Figure 5C). Twelve metabolites were identified as susceptibility biomarkers: rutin, 5-aminopentanoate, gallocatechin, nictoflorin, leucine, calycosin, phenylalanine, pinocembrin, glutamine, calycosin isomer, GlcNAc, and asparagine. Of these, five—rutin, gallocatechin, calycosin, pinocembrin, and calycosin isomer—were also selected in the original comparison (Figure 5C).

**Figure 5:**
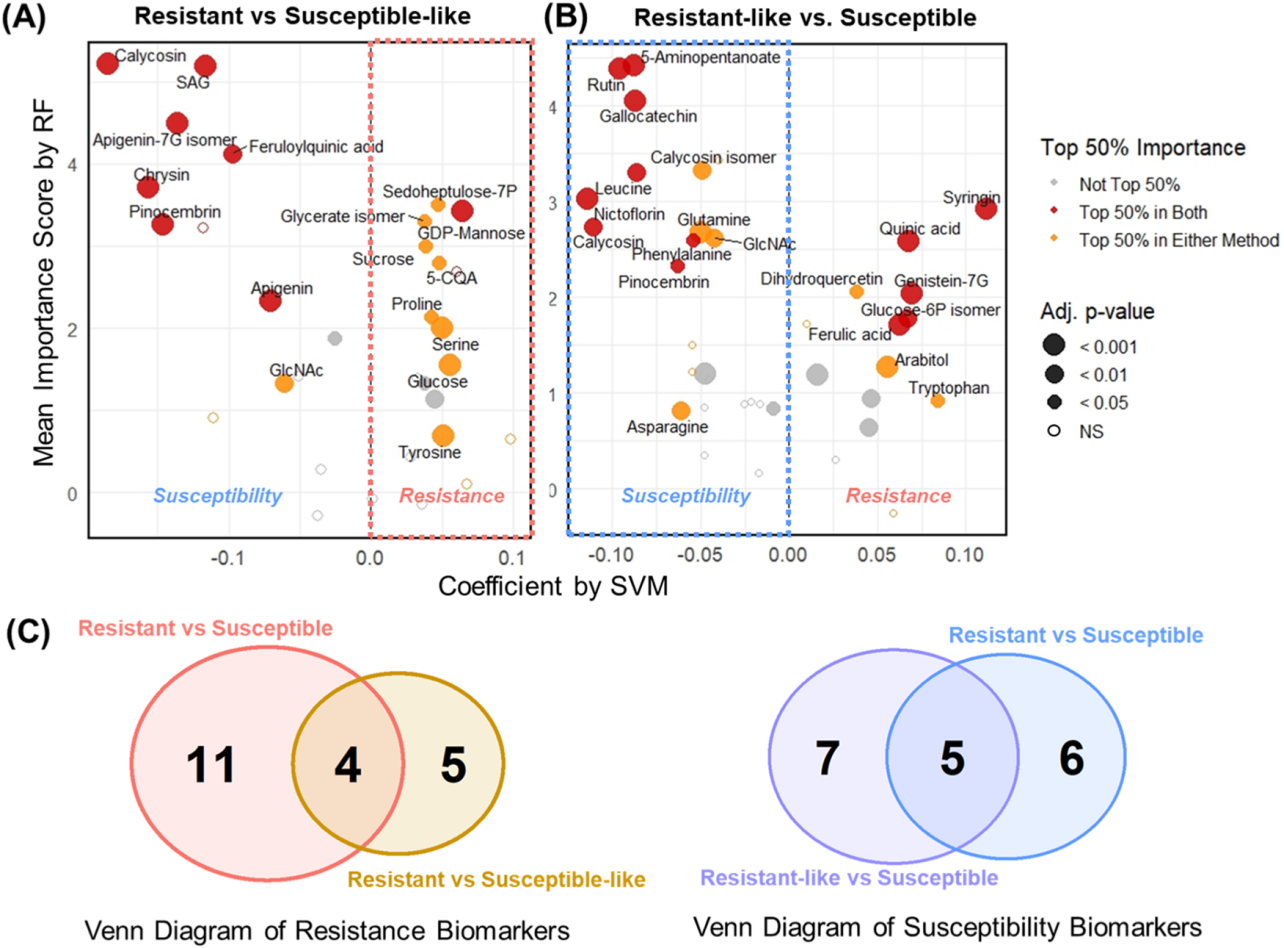
Identification of additional scab-resistance and -susceptibility biomarkers using subgroup comparisons. Significantly important metabolites by machine learning algorithms between (A) resistant and susceptible-like cultivars and between (B) resistant-like and susceptible cultivars. On the *x*-axis, positive SVM coefficients indicate association with the resistance group; negative coefficients indicate association with the susceptible group. On the *y*-axis, RF importance scores reflect the decrease in classification accuracy when a given metabolite is excluded; higher values denote greater contribution to model performance. Dot color represents whether the metabolite ranked in the top 50% of importance in either or both methods. Dot size reflects statistical significance based on linear regression; larger dots indicate lower FDR-adjusted *p*-values. Metabolites with no significant difference between groups are represented by hollow circles (NS, not significant, *p* ≥ 0.05). (C) Venn diagrams showing the number of overlapping biomarkers identified from subgroup comparisons with those from the original resistant vs susceptible classification. Left: comparison of resistance biomarkers. Right: comparison of susceptibility biomarkers.

In total, 20 compounds were consolidated as scab resistance biomarkers and 18 as susceptibility biomarkers (Figure 5C). Standardized effect sizes (Cohen’s *d*) summarizing differences in log_2_ transformed metabolite abundances across resistance categories are shown in Figure 6 and Supporting Table S5. Effect sizes for resistance biomarkers were calculated relative to the susceptible group, whereas susceptibility biomarkers were calculated relative to the resistant group, as these reference groups exhibited the lowest abundance for their respective biomarker sets. Resistance biomarkers displayed progressively increasing effect sizes from the susceptible to the resistant category, with most exhibiting medium to large magnitudes (|*d*| > 0.5), indicating strong and directionally consistent metabolic shifts associated with resistance. Conversely, susceptibility biomarkers showed increasing effect sizes toward the susceptible group, consistent with their accumulation in susceptible cultivars. The majority of susceptibility biomarkers also exceeded the medium-effect threshold, reflecting robust and biologically meaningful differences aligned with resistance levels.

**Figure 6:**
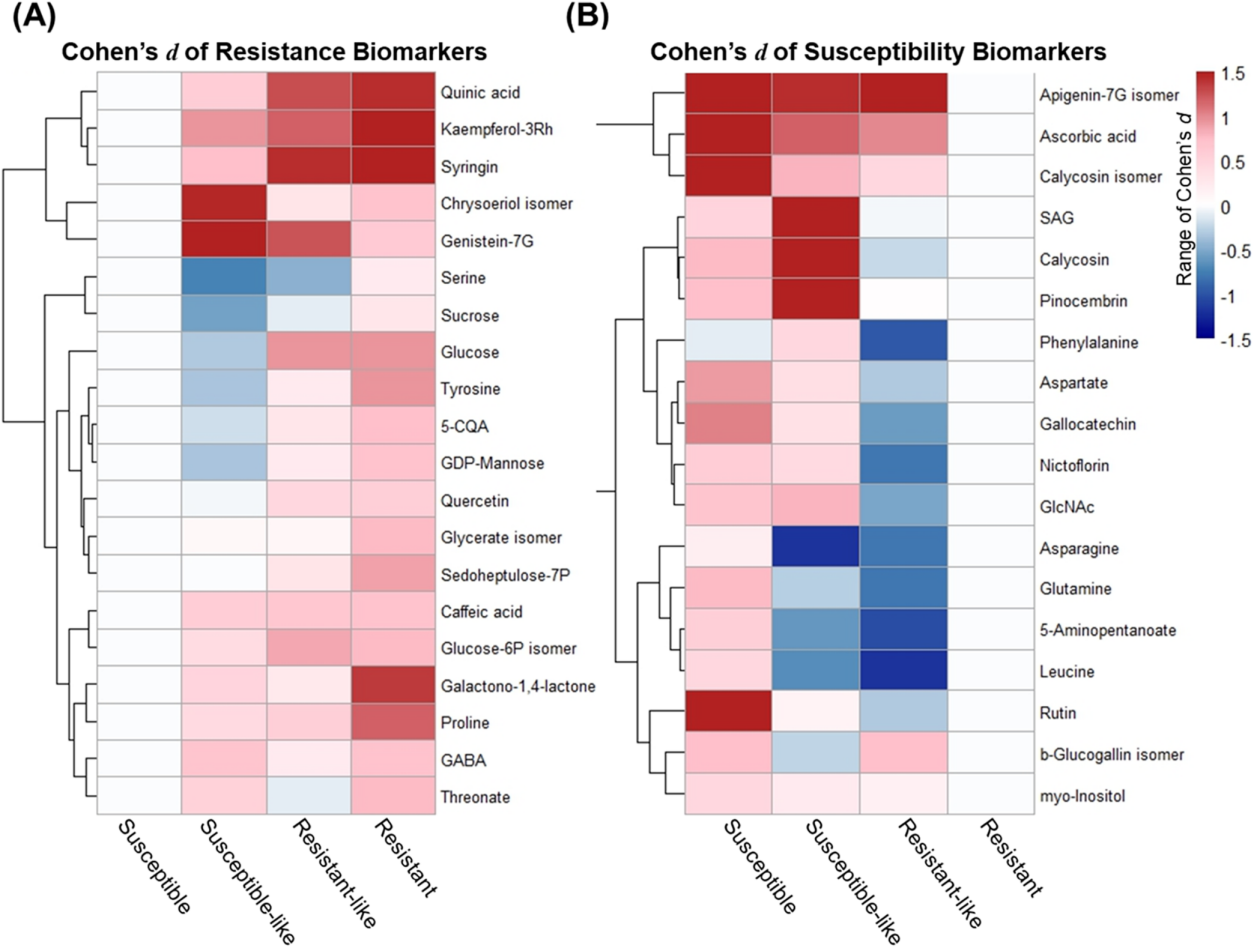
Standardized effect-size heatmaps (Cohen’s *d*) illustrating metabolite differences across pecan resistance groups. (A) Susceptibility biomarkers are shown relative to the resistant group, which is expected to exhibit the lowest abundance of these compounds. (B) Resistance biomarkers are shown relative to the susceptible group. Cohen’s *d* was calculated from replicate-level log_2_ transformed intensities using the pooled standard deviation estimator. Heatmaps use a symmetric color scale (−1.5 to +1.5) with a five-color palette (“darkblue”, “steelblue”, “white”, “pink”, “firebrick”), where nonwhite colors indicate deviating abundance relative to the reference group. Rows represent individual biomarkers, and columns correspond to four scab-resistance groups.

### 3.5 Metabolic Mechanisms Underlying Resistance and Susceptible Biomarkers

#### 3.5.1 Variation in Secondary Metabolism between Scab-Resistant and Susceptible Pecan Trees

Secondary metabolism plays a central role in plant defense, particularly through the production of various flavonoids and phytohormones with antifungal properties.^28,29,39^ To explore the metabolic mechanisms differentiating resistant and susceptible pecan trees, a secondary metabolism map was constructed based on the identified biomarkers (Figure 7).

**Figure 7:**
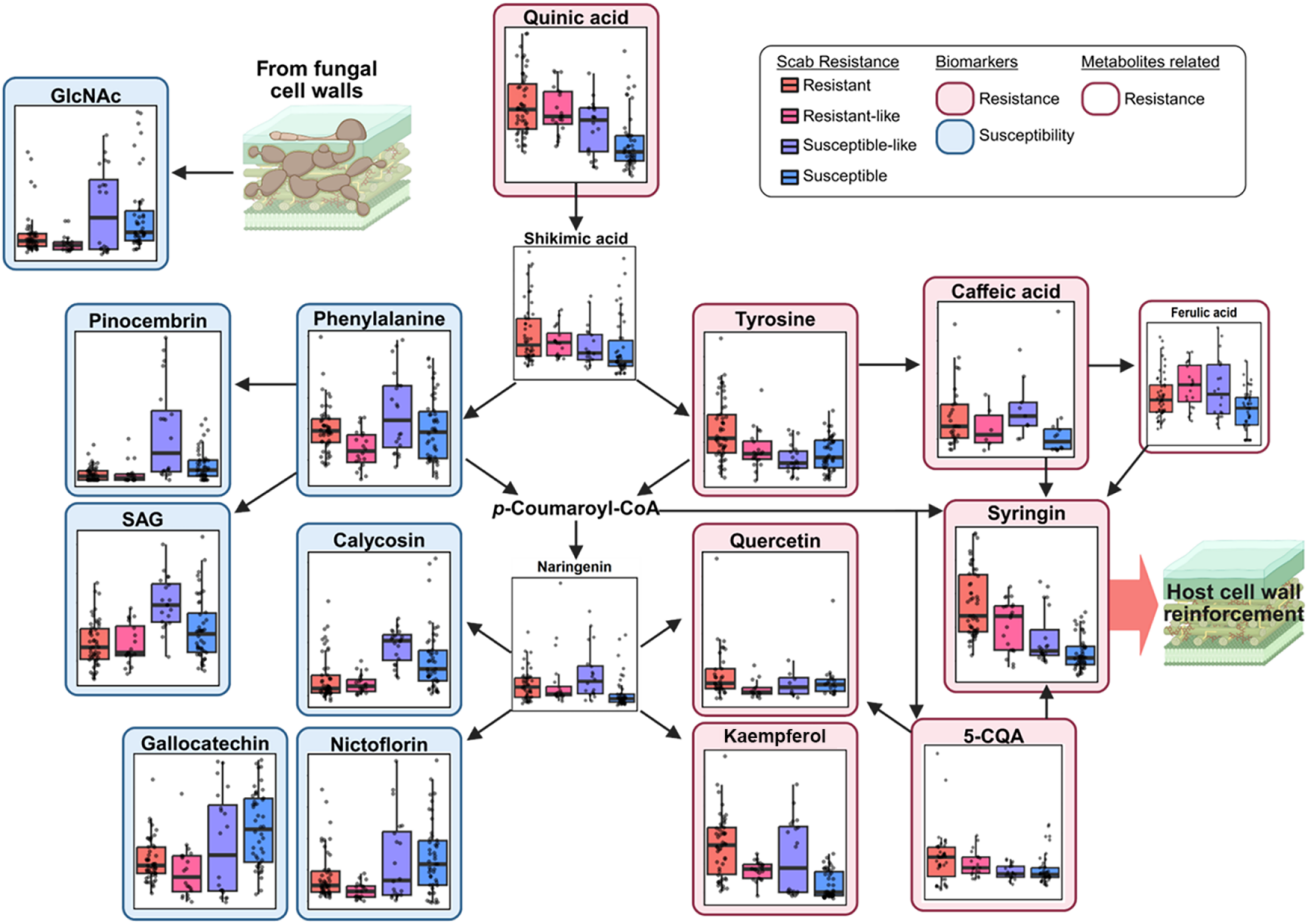
Pathway-based mapping of secondary metabolism in mature pecan trees with varying levels of resistance, based on identified biomarkers. Colored borders indicate biomarker associations: red tone represents resistance, and blue tone represents susceptibility. “Biomarkers” refer to metabolites that were both statistically significant (*p* < 0.05) and highly ranked by ML classifiers, while “Metabolites related” met the significance threshold but were not top-ranked in ML classification. Boxplots show normalized metabolite abundance across four scab-resistance groups: Resistant, resistant-like, susceptible-like, and susceptible.

One of the top-ranked resistance biomarkers, syringin—a glycosylated precursor of lignin—was elevated in the resistant group (resistant and resistant-like) and showed a decreasing trend in the susceptible group (susceptible and susceptible-like). Lignin contributes to structural defense by reinforcing cell walls and limiting pathogen invasion.^40^ Several upstream metabolites of syringin in the phenylpropanoid pathway, such as 5-CQA, caffeic acid, and tyrosine, were also upregulated in the resistant group. These results align with prior transcriptomic study comparing the resistant cultivar “Elliott” and the susceptible cultivar “Pawnee“, which found upregulation of lignin biosynthesis genes in resistant genotypes.^11^ Similar associations between lignin accumulation and disease resistance have been reported in other crops, including pigeon pea,^41^ tobacco,^42^ and jujube fruit.^43^ While lignification is part of normal leaf maturation, contributing to baseline physical defense against pathogens in pecan trees,^5,44^ our result suggests this process may be more pronounced in resistant genotypes, even in the absence of infection. Exogenous application of caffeic acid has been shown to enhance fungal resistance in pear fruit^45^ and apples^46^ through activation of the phenylpropanoid pathway, potentially leading to increased downstream lignin biosynthesis. Together, these findings suggest that lignin biosynthesis intermediates have potential both as metabolic markers of resistance and as candidate targets for resistance enhancement strategies in pecan.

Consistent with prior metabolomics work in mature pecan leaves,^19^ the flavonoid quercetin was again identified as a resistance-associated biomarker. However, several other flavonoids, such as calycosin, nictoflorin, and gallocatechin, which share the same biosynthetic precursor, were instead associated with susceptibility. This contradiction may reflect the complexity of plant defense regulation under natural field conditions, where both resistant and susceptible trees are continuously exposed to various pathogens including *V. effusa*. In such environments, susceptible trees may also upregulate flavonoid biosynthesis as part of a generalized stress response. This interpretation is supported by previous transcriptomic research showing that R-genes, responsible for pathogen recognition and defense activation, were expressed in both scab-resistant and -susceptible trees.^20^ Similar patterns have been observed in other crops; for example, in apple scab, resistance remains effective even when flavonol levels are low in resistant apple genotypes.^47^ Altogether, the evidence indicates that flavonoids alone may not reliably predict resistance. Rather, the timing and effectiveness of flavonoid-related responses, likely governed by genotype-specific regulatory mechanisms, may play a more critical role in determining the outcome of plant-pathogen interactions.

Salicylic acid (SA) is a key immune-related phytohormone,^48,49^ often stored in the form of salicylate 2-O-β-d-glucoside (SAG) to avoid cytotoxicity.^50^ While free SA was detected below the limit of quantification in our samples—likely due to the absence of active infection—SAG was quantified and identified as a susceptibility biomarker. This finding aligns with a previous transcriptomic study showing downregulation of *MPK9*, a positive regulator of SA signaling, in scab-resistant pecan trees compared to susceptible ones.^20,51^ Although SA is generally recognized as an immediate defense molecule in plants,^52^ excessive accumulation can be detrimental to cellular function and plant development.^53^ The lower SAG levels in resistant trees could be associated with differences in SA metabolism, though the functional implications remain unclear. Conversely, elevated SAG in susceptible trees might result from delayed or dysregulated SA signaling, possibly in response to latent infection. Supporting this assumption, our prior metabolomics work revealed a delayed but substantial increase in free SA in the scab-susceptible reaction model after infection, suggesting possible differences in the timing of defense responses (unpublished data, 2025).

GlcNAc, identified as a susceptibility biomarker, is a component of a fungal-derived pathogen-associated molecular pattern (PAMP) and is released during chitin degradation.^54^ Elevated GlcNAc in susceptible trees may indicate either a higher fungal burden or a reduced capacity for PAMP uptake and signaling. Further studies, such as proteomic or transcriptomic analysis regarding Chitinase activity and PAMP receptor expression, are needed to determine whether this signal reflects pathogen load or immune signaling efficiency.

#### 3.5.2 Variation in Primary Metabolism between Scab-Resistant and Susceptible Pecan Trees

Pathogen infection often triggers reprogramming of primary metabolism, particularly in pathways related to energy production, amino acid biosynthesis, and nitrogen assimilation—processes essential for initiating and sustaining defense responses.^27,55^ To investigate how these regulatory patterns differ between scab-resistant and susceptible pecan trees, a primary metabolism map was constructed based on the identified biomarkers (Figure 8).

**Figure 8:**
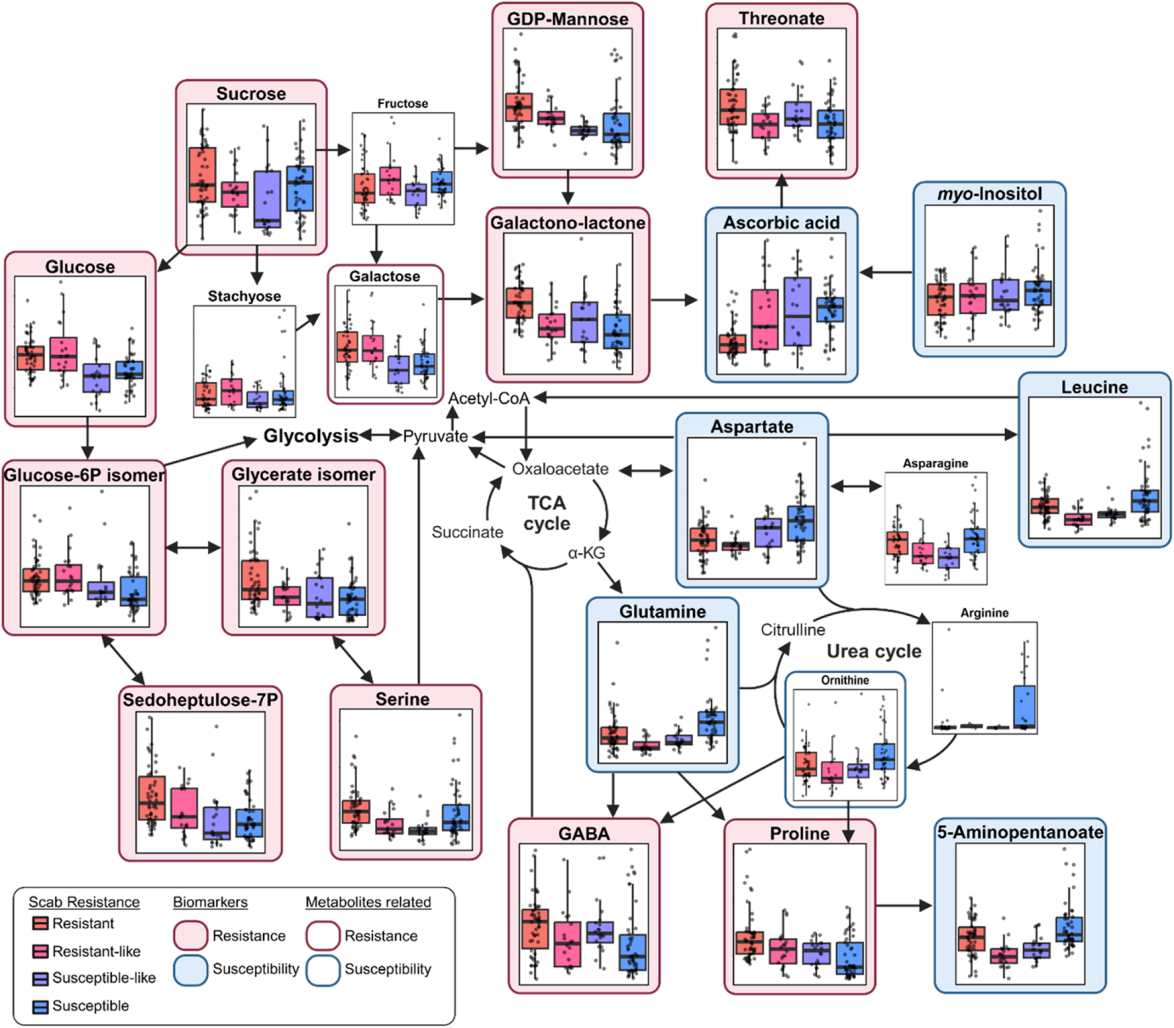
Pathway-based mapping of primary metabolism in mature pecan trees with varying levels of resistance based on identified biomarkers. Colored borders indicate biomarker associations: red tone represents resistance, and blue tone represents susceptibility. “Biomarkers” refer to metabolites that were both statistically significant (*p* < 0.05) and highly ranked by ML classifiers, while “Metabolites related” met the significance threshold but were not top-ranked in ML classification. Boxplots show normalized metabolite abundance across four scab-resistance groups: Resistant, resistant-like, susceptible-like, and susceptible.

Resistant cultivars exhibited significantly higher levels of key metabolites involved in energy-generating pathways such as glycolysis and pentose phosphate pathway. These included glucose-6P isomer, glycerate isomer, sedoheptylose-7P, glucose, galactose, and sucrose. In contrast, susceptible cultivars showed elevated levels of nitrogen-transport amino acids such as aspartate and glutamine, which are known to serve as primary nitrogen sources for fungal pathogens.^56^ Pathogens typically acquire nutrients from the host leaf apoplast, facilitating their growth and virulence.^57,58^ Susceptible trees also displayed higher levels of the urea cycle intermediate ornithine, further supporting the generation of a nitrogen-rich environment. This metabolic divergence suggests that susceptible trees may create conditions conducive to fungal growth, whereas resistant trees appear to reallocate metabolic resources toward energy production and readiness for more effective defense activation.^30^ This distinct energetic allocation likely underpins their differing levels of scab resistance.

Proline and γ-aminobutyric acid (GABA)—derived from glutamine and ornithine—were significantly enriched in resistant trees and identified as biomarkers by both ML models. Proline functions as a reactive oxygen species (ROS) scavenger^59,60^ and also contributes to structural defense via incorporation into proline-rich cell wall proteins.^61^ GABA has been implicated in enhanced disease resistance in several crops, including apple against *Penicillium expansum* ^62^ and tomato against *Alternaria alternata*,^63^ following exogenous GABA application. Although susceptible trees had higher levels of precursor amino acids such as glutamine and ornithine, their metabolic allocation appeared less oriented toward the synthesis of downstream defense-related compounds. This may be partly explained by differential activity of aspartate aminotransferase (AspAT). In *Arabidopsis thaliana*, overexpression of AspAT genes has been associated with increased pathogen susceptibility due to enhanced synthesis of nitrogen-rich amino acids that serve as nutrient sources for pathogens, which may coincide with lower levels of glutamate-derived compounds such as proline and GABA.^64^ While a similar pattern in pecan cultivars is possible, further investigation is needed to determine whether the observed differences in amino acid profiles reflect differences in nitrogen allocation strategies or other metabolic processes between resistant and susceptible genotypes.

In the primary metabolism, ascorbic acid was identified as a top-ranked susceptibility biomarker. While ascorbic acid is an antioxidant involved in plant stress responses,^65^ its elevated abundance in susceptible trees does not appear to reflect effective defense signaling.^66^ Major precursors (galactose, GDP-mannose, and galactono-1,4-lactone) of ascorbic acid in the Smirnoff–Wheeler pathway^67^ were identified as resistance markers, being significantly more abundant in resistant trees. This suggests a more proactive and readily available capacity for ascorbate biosynthesis upon infection. Resistant trees also exhibited higher levels of threonate, a major product of ascorbic acid degradation,^68^ which might indicate active turnover and utilization of ascorbate in ROS detoxification. Similar to SA, our prior study observed delayed accumulation of ascorbic acid in the scab-susceptible response model following infection (unpublished, 2025). These imply that the timing and efficiency of ascorbic acid activation may be critical for distinguishing levels of scab resistance.

In this study, orchard-grown mature pecan trees were investigated to identify biomarkers of scab resistance and susceptibility using a pathway-based metabolomics integrated with machine learning algorithms. Resistant cultivars exhibited metabolic preparedness, with increased levels of precursors involved in lignin biosynthesis, energy metabolism, and antioxidant turnover. In contrast, susceptible cultivars showed potential signs of metabolic imbalance, accumulating defense-related end-products and nitrogen-rich compounds while exhibiting limited precursor availability, suggesting reduced flexibility in their response to *V. effusa*. These findings reveal distinct metabolic features underlying scab resistance in pecans, which can enhance our understanding of core defense mechanisms. The biomarkers identified here reflect constitutive metabolic signatures associated with resistance phenotypes rather than active defense mechanisms during pathogen challenge. This distinction is crucial for biological interpretation, as these markers indicate metabolic states that correlate with resistance outcomes but do not directly demonstrate causal defense pathways or mechanisms of pathogen inhibition. The identified biomarkers may support the breeding of scab-resistant cultivars, as well as inform exogenous application strategies aimed at priming defense pathways, ultimately contributing to more sustainable disease management in pecan orchards.

## Supplementary Material



## Data Availability

All relevant data in this study are provided in the article and its Supporting Information.

## Notes

Information on 171 target metabolites, their analytical conditions, and identification methods; results of biomarker selection by machine learning algorithms for classifying between resistant and susceptible mature trees, and workflow of biomarker identification using machine learning (PDF)

## Abbreviations Used

ML: machine learning
LRR: leucine-rich repeat
MAPKs: mitogen-activated protein kinases
ADP: adenosine 5′-diphosphate
GTP: guanosine-5′-triphosphate
GDP-mannose: guanosine 5′-diphosphate-d-mannose
GABA: γ-aminobutyric acid
AMP: adenosine 5′-monophosphate
ATP: adenosine 5′-triphosphate
kaempferol-3Rh: kaempferol 3-*O*-α-L-rhamnoside
Apigenin-7G: apigenin 7-*O*-glucoside
DHAP: dihydroxyacetone phosphate
DMAPP: dimethylallyl diphosphate
GAD-3P: glyceraldehyde 3-phosphate
GDP: guanosine 5′-diphosphate
GMP: guanosine 5′-monophosphate
Trifolin: kaempferol 3-*O*-galactoside
Nictoflorin: kaempferol 3-*O*-rutinoside
GlcNAc: *N*-acetyl-d-glucosamine
PEP: phosphoenol pyruvic acid
Rutin: quercetin 3-*O*-rutinoside
Sedoheptulose-7P: sedoheptulose 7-phosphate
UDP-glucose: uridine diphosphate glucose
5-CQA: 5-caffeoylquinic acid
SAG: salicylate 2-*O*-β-d-glucoside
SA: salicylic acid
LC-MS: liquid chromatography–mass spectrometry
UPLC: ultraperformance liquid chromatography
HILIC: hydrophilic interaction chromatography
RP: reverse-phase
QC: quality control
QqQ: triple quadrupole
DMRM: dynamic multiple reaction monitoring
ESI: electrospray ionization
RSD: relative standard deviation
PCA: principal component analysis
TCA cycle: tricarboxylic acid cycle
SVM: support vector machine
RF: random forest
FDR: false discovery rate
MCC: matthews correlation coefficient
TP: true positive
FP: false positive
TN: true negative
FN: false negative
RMSE_cv_: root-mean-square error by cross validation
