# Supplementary material for: Integrative Metabolomics and Machine Learning Approach to Uncover Scab Resistance Markers in Mature Pecan Trees
Source: J Agric Food Chem. 2026 Apr 29;74(18):14619–33. doi: 10.1021/acs.jafc.5c16064 (PMC13178070; doi:10.1021/acs.jafc.5c16064)

Supplementary Materials

Integrative Metabolomics and Machine Learning Approach to Uncover Scab Resistance Markers in Mature Pecan Trees

Min Jeong Kang[a], Ronald B. Pegg[a], William L. Kerr[a], M. Lenny Wells[b], Patrick J. Conner[b], Joon Hyuk Suh[a,*]

[a]*Department of Food Science and Technology, College of Agricultural and Environmental Sciences, University of Georgia, 100 Cedar Street, Athens, GA 30602, USA*

[b]*Department of Horticulture, College of Agricultural and Environmental Sciences, University of Georgia, 2360 Rainwater Road, Tifton, GA 31793, USA*

*Corresponding Author:

Joon Hyuk Suh, Ph.D.

Department of Food Science and Technology

College of Agricultural and Environmental Sciences

University of Georgia

100 Cedar Street, Athens, GA 30602, USA

Tel.: +1-706-542-7909 E-mail: J.Suh@uga.edu

TABLE OF CONTENTS

Page

Supplementary Table S1. Information on 171 target metabolites, their analytical conditions, and identification methods S-3

Supplementary Table S2. Evaluation of batch reliability using pooled QC samples S-10

Supplementary Table S3. Results of biomarker selection by machine learning algorithms for classifying between resistant and susceptible mature trees S-13

Supplementary Table S4. Results of biomarker selection by machine learning algorithms for classifying between resistant vs susceptible-like and resistant-like vs susceptible groups of mature trees in pecan leaflets S-16

Supplementary Table S5. Cohen's *d* values summarizing standardized differences in log_2 transformed metabolite abundances across scab resistance groups S-19

Supplementary Figure S1. Workflow of biomarker identification using machine learning ... S-21

Supplementary Figure S2. Permutation test results of classification between resistant vs susceptible using support vector machine and random forest S-22

Supplementary Table S1. Information on 171 target metabolites, their analytical conditions, and identification methods.

Compound class	Compound name	Polarity	Precursor (m/z)	Product (m/z)	Retention time (min)	Collision Energy (V)	Frag. Energy (V)	Identification	
								Column[a]	Standard[b]
Sugars/ Sugar acids	Arabinose	Negative	149.0	89.0	3.9	10	100	HILIC	O
	Arabitol	Negative	151.1	89.1	3.3	10	88	HILIC	O
	Fructose	Negative	179.1	89.1	3.4	5	50	HILIC	O
	Galactose	Negative	179.1	89.1	4.1	5	50	HILIC	O
	Glucose	Negative	179.1	89.1	3.8	5	50	HILIC	O
	Raffinose	Negative	503.2	179.1	8.6	22	80	HILIC	O
	Ribose	Negative	149.0	59.1	2.7	14	80	HILIC	O
	Sorbitol	Negative	181.1	89.1	4.0	13	102	HILIC	O
	Stachyose	Negative	665.2	383.1	9.8	34	80	HILIC	O
	Sucrose	Negative	341.1	179.1	6.0	14	128	HILIC	O
	Trehalose	Negative	341.1	179.1	7.4	14	128	HILIC	O
	Xylose	Negative	149.0	59.1	3.1	14	80	HILIC	O
Amino acids	Alanine	Positive	90.2	44.2	6.2	15	70	HILIC	O
	Arginine	Positive	175.1	70.1	10.9	29	110	HILIC	O
	Asparagine	Positive	133.1	74.1	7.4	17	76	HILIC	O
	Aspartate	Positive	134.0	74.1	8.7	15	102	HILIC	O
	Citrulline	Positive	176.1	70.1	8.1	25	100	HILIC	O
	Glutamic acid	Positive	148.1	84.1	8.9	18	76	HILIC	O
	Glutamine	Positive	147.1	84.1	7.3	17	80	HILIC	O
	Hypoxanthine	Positive	137.0	110.0	2.3	25	102	HILIC	O
	Isoleucine	Positive	132.1	86.1	3.4	9	76	HILIC	O
	Leucine	Positive	132.1	86.1	3.2	9	76	HILIC	O
	Lysine	Positive	147.1	84.2	10.8	18	76	HILIC	O
	Methionine	Positive	150.1	104.0	3.6	9	80	HILIC	O
	Ornithine	Positive	133.1	70.2	10.8	21	80	HILIC	O
	Phenylalanine	Positive	166.1	102.9	2.9	38	80	HILIC	O
	Proline	Positive	116.1	70.2	4.6	17	76	HILIC	O

	Serine	Positive	106.1	60.2	7.4	13	76	HILIC	O
	Tryptophan	Positive	205.1	188.1	3.2	10	80	HILIC	O
	Tyrosine	Positive	182.1	91.0	4.0	13	76	HILIC	O
	Valine	Positive	118.1	72.2	4.2	12	76	HILIC	O
Organic acids	2,5-Dihydroxybenzoic acid	Negative	153.0	108.0	10.0	25	80	RP	O
	α-Ketoglutaric acid	Negative	145.0	101.1	8.5	5	76	HILIC	O
	Ascorbic Acid	Negative	175.0	114.8	2.6	8	100	RP	O
	Benzoic acid	Negative	121.1	77.1	12.7	10	120	RP	O
	Caffeic acid	Negative	179.0	135.0	10.2	17	102	RP	O
	Caffeoyl shikimic acid (CSA)	Negative	335.0	179.0	10.2	17	102	RP	X (Jaiswal, Sovdat, Vivan, & Kuhnert, 2010)
	5-*O*-caffeoylquinic acid (5-CQA)	Positive	355.1	163.0	9.3	13	76	RP	O
	4-*O*-caffeoylquinic acid (4-CQA)	Positive	355.1	163.0	9.6	13	76	RP	X (Ripper et al., 2022)
	3-*O*-caffeoylquinic acid (3-CQA)	Positive	355.1	163.0	8.9	13	76	RP	X (Ripper et al., 2022)
	Cinnamic acid	Positive	149.1	65.1	21.9	29	170	RP	O
	Citric acid	Negative	191.0	111.1	3.0	10	100	RP	O
	Ferulic acid	Negative	193.1	134.0	2.4	17	102	HILIC	O
	Fumaric acid	Negative	115.0	71.1	9.4	5	76	HILIC	O
	γ-Aminobutyric acid (GABA)	Positive	104.1	87.1	8.0	10	88	HILIC	O
	Gallic acid	Negative	169.0	125.0	8.3	13	102	RP	O
	Glucuronate	Negative	193.0	113.1	8.0	9	76	HILIC	O
	Glycerate	Negative	105.0	75.2	4.5	9	80	HILIC	O
	Glycerate isomer	Negative	105.0	75.2	4.3	9	80	HILIC	X
	Glycolate	Negative	75.0	47.0	4.1	12	100	HILIC	O
	Isocitric acid	Negative	191.0	173.0	3.6	5	80	RP	O
	Maleic acid	Negative	115.0	71.1	3.2	9	50	HILIC	O
	Malic acid	Negative	133.0	114.7	9.4	9	90	HILIC	O
	Malonic acid	Negative	103.1	59.1	9.0	5	50	HILIC	O

	Oxalate	Negative	89.0	61.1	9.8	10	50	HILIC	O
	p-Coumaric acid	Negative	163.0	119.1	11.6	14	102	RP	O
	Phosphoenolpyruvic acid (PEP)	Negative	167.0	79.1	10.3	13	154	HILIC	O
	Pyruvic acid	Negative	87.0	43.0	2.4	12	70	HILIC	O
	Quinic acid	Negative	191.1	85.1	4.8	25	128	HILIC	O
	Shikimic acid	Negative	173.0	93.1	7.5	21	102	HILIC	O
	Sinapic acid	Negative	223.1	208.0	2.3	13	80	HILIC	O
	Succinic acid	Negative	117.0	72.8	9.5	9	100	HILIC	O
	Tartaric acid	Negative	149.0	87.0	9.1	12	89	HILIC	O
	Threonate	Negative	135.0	89.1	4.7	9	76	HILIC	O
	Uric acid	Negative	167.0	124.0	4.1	14	120	HILIC	O
Nucleotides/ Nucleosides/ Sugar nucleotides	Adenine	Positive	136.1	119.0	2.1	25	128	HILIC	O
	Adenosine	Positive	268.1	136.0	2.1	19	102	HILIC	O
	Adenosine 5'-diphosphate (ADP)	Positive	428.0	136.1	9.7	29	102	HILIC	O
	Adenosine 5'-monophosphate (AMP)	Positive	348.1	136.0	9.2	17	102	HILIC	O
	AMP isomer	Positive	348.1	136.0	8.8	17	102	HILIC	X
	Adenosine 5′-triphosphate (ATP)	Positive	508.0	136.0	10.0	33	128	HILIC	O
	ATP isomer	Positive	508.0	136.0	10.4	33	128	HILIC	X
	Deoxyadenosine	Positive	252.1	136.0	2.0	13	76	HILIC	O
	Guanosine 5′-diphosphate (GDP)	Positive	444.0	152.0	2.5	17	50	RP	O
	GDP-mannose	Positive	606.1	152.0	9.7	33	102	HILIC	O
	Guanosine 5′-monophosphate (GMP)	Positive	364.1	152.0	9.9	17	76	HILIC	O
	Guanosine-5'-triphosphate (GTP)	Positive	524.0	152.0	10.3	25	128	HILIC	O
	Guanosine	Positive	284.1	110.0	3.0	50	76	HILIC	O
	Inosine	Positive	269.1	119.0	2.7	50	50	HILIC	O
	Uridine diphosphate glucose (UDP-glucose)	Negative	565.1	323.1	9.0	25	180	HILIC	O
	UDP-glucuronic acid	Negative	579.0	402.8	9.2	32	180	HILIC	O
Flavonoids	Apigenin	Positive	271.1	119.0	19.6	37	170	RP	O
	Apigenin 7-*O*-Glucoside	Positive	433.1	271.1	13.0	29	80	RP	O
	Biochanin A	Negative	283.1	268.1	25.2	25	140	RP	O

Brassinolide	Positive	481.4	71.2	24.3	37	110	RP	O
Calycosin	Positive	285.1	270.1	16.4	25	140	RP	O
Calycosin isomer	Positive	285.1	270.1	15.8	25	140	RP	X
Calycosin 7-*O*-glucoside	Positive	447.1	225.1	11.1	50	102	RP	O
Catechin	Negative	289.1	245.1	9.6	13	128	RP	O
Catechin isomer	Negative	289.1	245.1	10.1	13	128	RP	X
Chrysin	Positive	255.1	153.0	24.3	37	128	RP	O
Chrysoeriol	Positive	301.1	286.1	20.1	29	140	RP	O
Chrysoeriol isomer	Positive	301.1	286.1	19.8	29	140	RP	X
Daidzein	Positive	255.1	199.0	15.6	29	140	RP	O
Dihydrokaempferol	Negative	287.0	259.1	14.1	13	102	RP	O
Dihydromyricetin	Negative	319.0	193.1	10.7	9	128	RP	O
Dihydroquercetin (Taxifolin)	Negative	303.0	285.1	12.4	10	102	RP	O
Epigallocatechin gallate (EGCG)	Negative	457.1	169.0	10.2	17	128	RP	O
Eriodictyol	Negative	287.0	151.0	15.8	13	154	RP	O
Formononetin	Positive	269.1	197.0	22.0	45	140	RP	O
Gallocatechin	Negative	305.1	125.0	8.8	21	128	RP	O
Genistein	Positive	271.1	91.1	18.9	45	154	RP	O
Genistein 7-*O*-glucoside (Genistein-7G)	Positive	433.1	271.1	12.9	17	102	RP	O
Kaempferol	Negative	285.0	195.1	20.3	33	154	RP	O
Kaempferol 3-*O*-galactoside (Trifolin)	Positive	449.1	287.1	12.5	13	80	RP	O
Kaempferol 3-*O*-glucoside (Astragalin)	Positive	449.1	287.1	12.9	13	80	RP	O
Kaempferol 3-*O*-α-L-rhamnoside (Kaempferol-3Rh)	Positive	433.1	287.1	14.1	9	80	RP	O
Kaempferol 3-*O*-rutinoside (Nictoflorin)	Positive	595.2	287.0	12.3	21	110	RP	O
Luteolin	Positive	287.1	153.0	17.1	37	128	RP	O
Luteolin-glucuronide	Negative	461.1	285.1	12.0	25	140	RP	O
Luteolin 7-*O*-glucoside (luteoloside)	Negative	447.1	285.1	11.8	29	200	RP	O
Naringenin	Positive	273.1	153.0	17.9	29	128	RP	O

	Naringenin chalcone	Positive	273.1	153.0	17.8	29	128	RP	O
	Naringenin 7-*O*-glucoside (prunin)	Positive	433.0	271.1	13.0	21	80	RP	O
	Naringin	Negative	579.2	271.1	12.3	37	200	RP	O
	Pinobanksin	Negative	271.1	253.1	18.1	25	128	RP	O
	Pinocembrin	Negative	255.1	213.1	23.4	21	128	RP	O
	Prunetin	Positive	285.1	167.0	25.5	33	128	RP	O
	Prunetin isomer	Positive	285.1	167.0	25.2	33	128	RP	X
	Quercetin	Negative	301.0	151.0	17.3	21	128	RP	O
	Quercetin 3-*O*-glucoside (Isoquercetin)	Positive	465.1	303.1	11.9	13	80	RP	O
	Quercetin 3-*O*-rhamnoide (Quercitrin)	Positive	449.1	85.1	12.8	25	80	RP	O
	Quercetin 3-*O*-rutinoside (Rutin)	Negative	609.1	301.1	11.4	37	200	RP	O
	Quercetin 3-*O*-sophoroside (Baimaside)	Negative	625.1	271.1	10.3	50	200	RP	O
	Rhoifolin	Positive	579.2	271.0	11.0	33	140	RP	O
	Sakuranetin	Negative	285.1	165.0	23.5	17	102	RP	O
	Sissotrin	Positive	447.1	152.0	16.4	50	110	RP	O
	Tricetin	Positive	303.1	153.0	14.0	41	180	RP	O
	Tricetin-glucoside (Tricetin-G)	Positive	465.0	303.1	11.9	41	180	RP	X
	Tricin	Negative	329.1	299.1	20.6	33	128	RP	O
	Vestitone	Positive	287.1	163.0	16.5	17	110	RP	O
Plant hormones	Abscisic acid (ABA)	Negative	263.1	153.1	14.6	10	120	RP	O
	Indole-3-acetic acid (IAA)	Positive	176.1	130.1	13.8	17	80	RP	O
	Indole-3-carboxylic acid	Negative	160.0	116.1	12.6	17	80	RP	O
	Jasmonic acid-isoleucine (JA-Ile)	Positive	324.2	86.1	19.9	29	80	RP	O
	Jasmonic acid	Negative	209.1	59.2	17.0	13	76	RP	O
	Salicylic acid 2-*O*-β-D-glucoside (SAG)	Negative	299.1	137.0	9.0	21	76	RP	O
	SAG isomer	Negative	299.1	137.0	8.3	21	76	RP	X
	Salicylic acid	Negative	137.0	93.0	13.7	17	80	RP	O
	trans-zeatin (Tzeatin)	Positive	220.1	136.1	8.4	17	110	RP	O
	trans-zeatin riboside (TzeatinR)	Positive	352.2	220.1	8.9	21	110	RP	O

Others	3-Phosphoglycerate	Positive	187.0	105.0	7.0	5	76	HILIC	O
	5-Aminopentanoate	Positive	118.1	101.1	8.3	9	76	HILIC	O
	Acetyl-L-lysine	Positive	189.1	84.1	7.0	25	102	HILIC	O
	β-Glucogallin	Negative	331.1	169.0	8.1	21	110	RP	O
	β-Glucogallin isomer	Negative	331.1	169.0	8.5	21	110	RP	X
	cis-Aconitate	Negative	173.0	129.0	9.7	5	76	HILIC	O
	Coniferyl alcohol	Negative	179.1	146.0	20.4	25	180	RP	O
	Diacetylchitobiose	Positive	425.2	204.1	5.8	13	154	HILIC	O
	Dehydroascorbate	Negative	173.0	143.0	2.1	5	50	HILIC	O
	Dihydroxyacetone phosphate (DHAP)	Negative	169.0	97.0	7.8	10	120	HILIC	O
	Dimethylallyl diphosphate (DMAPP)	Positive	247.0	124.1	2.6	13	80	RP	O
	Galactono-1,4-lactone	Negative	177.0	159.1	2.4	5	102	HILIC	O
	Galangin	Positive	271.1	69.1	26.0	50	154	RP	O
	Glucosamine	Positive	180.1	72.1	6.0	17	76	HILIC	O
	Glucose 6-phosphate	Negative	259.0	97.0	10.0	15	102	HILIC	O
	Glucose 6-phosphate isomer	Negative	259.0	97.0	9.7	15	102	HILIC	X
	Glyceraldehyde 3-phosphate	Negative	169.0	97.0	5.0	50	7.8	HILIC	O
	trans-4-Hydroxy-L-proline	Positive	132.1	86.1	6.1	13	80	HILIC	O
	Methyl-2-oxobutyric acid	Positive	268.1	136.0	8.0	17	102	RP	O
	myo-Inositol	Negative	179.1	161.1	6.9	10	112	HILIC	O
	N-Acetyl-D-glucosamine (GlcNAc)	Positive	222.1	138.0	3.5	17	50	HILIC	O
	p-Coumaraldehyde	Positive	149.1	77.1	14.0	37	80	RP	O
	p-Coumaryl-alcohol	Negative	149.1	131.1	10.8	9	80	RP	O
	Podophyllotoxin (PPT)	Positive	415.1	397.4	20.0	15	132	RP	O
	Phosphoribosyl pyrophosphate (PRPP)	Positive	391.0	149.0	33.8	33	80	RP	O
	Salicin	Negative	285.1	123.1	2.1	9	110	HILIC	O
	Secoisolariciresinol	Negative	361.2	165.1	13.4	29	128	RP	O
	Sedoheptulose 7-phosphate (Sedoheptulose-7P)	Negative	289.0	97.0	9.8	21	102	HILIC	O
	Syringin	Positive	395.4	232.2	2.0	25	150	HILIC	O

	N,N',N''-Triacetylchitotriose	Positive	628.3	138.0	6.0	50	102	HILIC	O
Internal standards	Alanine-d_3	Positive	93.0	47.2	6.3	15	70	HILIC	O
	Apigenin-d_5	Positive	276.3	122.0	19.5	37	102	RP	O
	N,N-Dimethyl-d_6-glycine	Positive	110.0	64.2	4.5	17	90	HILIC	O
	Genistein-d_4	Positive	275.1	247.1	18.9	29	154	RP	O
	Hippuric acid-d_5	Negative	183.2	139.1	9.7	10	106	RP	O
	Methionine-d_3	Positive	154.0	57.2	3.6	15	100	HILIC	O
	Phenylalanine-$^{13}C_6$	Positive	172.2	126.1	2.9	10	92	HILIC	O
	Proline-d_3	Positive	119.0	73.2	4.6	18	92	HILIC	O
	Salicylic acid-d_4	Negative	141.1	97.0	13.6	25	140	RP	O

[a] Metabolites including internal standards were analyzed by two different columns: hydrophilic interaction chromatography (HILIC) and reverse-phase (RP).
[b] Commercially unavailable metabolites were identified by fragmentation patterns or reference papers.

Jaiswal, R.; Sovdat, T.; Vivan, F.; Kuhnert, N. Profiling and characterization by LC-MS_n of the chlorogenic acids and hydroxycinnamoylshikimate esters in Maté (*Ilex paraguariensis*). *J. Agric. Food Chem.* **2010**, *58* (9), 5471−5484. https://doi.org/10.1021/jf904537z.

Ripper, B.; Barreto, M. S.; Novaes, F. J. M.; de Godoy, M. G.; Freire, D. M. G.; de Rezende, C. M.; Fioresi, D. B.; Pinto, F. E.; Filgueiras, P. R.; Perrone, D. Comprehensive composition of flavor precursors in Kopi Luwak and Jacu exotic green bioprocessed coffees. *Front. Sustain. Food Syst.* **2022**, *6*, 824929. https://doi.org/10.3389/fsufs.2022.824929.

Supplementary Table S2. Evaluation of batch reliability using pooled QC samples.

Metabolite	RSD (%)		
	Batch 1	Batch 2	Batch 3
Acetyl-L-lysine	2.9	3.7	4.1
Adenine	2.9	6.6	11.1
Adenosine	3.9	6.4	9.9
ADP	4.3	5.3	8.0
Alanine	4.7	5.2	2.9
5-Aminopentanoate	3.0	5.2	11.8
AMP	4.4	12.0	13.5
AMP isomer	8.2	8.0	8.2
Apigenin	6.8	3.2	3.5
Apigenin-7G isomer	6.3	5.9	3.7
Arabinose	2.5	3.7	3.9
Arabitol	5.7	14.4	12.9
Arginine	1.3	2.6	4.4
Ascorbic acid	8.1	17.3	15.6
Asparagine	3.9	6.6	5.4
Aspartate	10.0	6.3	6.7
ATP	4.7	8.5	4.3
ATP isomer	9.6	20.6	22.3
β-Glucogallin	3.0	6.3	6.2
β-Glucogallin isomer	4.5	5.8	4.8
Biochanin A	6.0	7.9	6.4
Caffeic acid	10.6	10.9	8.9
Calycosin	7.9	3.0	1.7
Calycosin isomer	3.6	3.2	3.3
Catechin	2.7	5.2	2.1
Catechin isomer	4.8	6.0	7.1
Chrysin	4.0	2.2	3.1
Chrysoeriol isomer	4.3	4.2	3.6
cis-Aconitate	8.4	11.4	15.2
Citric acid	9.3	4.8	6.6
Citrulline	9.1	11.1	8.3
3-CQA	3.6	1.3	2.7
4-CQA	7.2	10.2	8.7
5-CQA	8.7	6.0	5.9
CSA	7.5	7.3	5.1
Dehydroascorbate	4.8	8.1	14.3
Deoxyadenosine	4.8	5.3	8.4
Dihydroquercetin	4.2	2.9	5.5
EGCG	6.8	5.5	4.6
Ferulic acid	11.3	8.7	22.0
Feruloylquinic acid	2.4	3.0	2.3
Fructose	3.8	4.7	7.6
Fumaric acid	3.6	3.3	5.4
GABA	2.9	6.5	2.7
Galactono-1,4-lactone	5.4	9.9	4.1
Gallic acid	10.9	5.2	15.9
Gallocatechin	2.1	6.0	3.8
GDP-mannose	5.4	4.9	4.7
Genistein-7G	5.1	3.7	4.6

Galactose	8.6	5.7	10.0
Glucose	4.1	7.4	5.6
Glucose-6P	6.7	15.8	11.1
Glucose-6P isomer	12.1	10.9	12.0
Glutamic acid	5.4	4.3	4.1
Glutamine	10.7	15.3	10.3
Glycerate isomer	4.2	5.7	4.9
Guanosine	4.9	3.3	6.5
trans-4-Hydroxy-L-proline	3.1	4.1	2.7
Inosine	4.3	8.4	8.5
Isoleucine	4.9	2.4	1.4
Trifolin	4.6	5.7	7.5
Astragalin	4.3	3.8	4.2
Kaempferol-3Rh	6.3	5.9	3.7
Nictoflorin	4.4	3.2	4.8
α-Ketoglutaric acid	3.1	3.8	4.4
Leucine	8.2	4.9	10.8
Lysine	1.1	3.1	1.9
Malic acid	3.1	3.5	3.3
Malonic acid	5.8	15.7	8.6
Methionine	2.9	6.8	6.2
Methyl-2-oxobutyric acid	6.4	2.2	2.8
myo-Inositol	5.8	4.2	6.0
GlcNAc	7.1	4.1	3.9
Naringenin	2.2	4.9	4.2
Prunin	5.6	3.7	9.4
Ornithine	7.4	2.4	1.6
p-coumaric acid	7.9	9.2	6.0
Phenylalanine	2.4	4.6	4.9
3-Phosphoglycerate	4.2	3.9	6.1
Pinocembrin	10.9	8.7	5.1
Proline	2.3	9.0	9.9
Prunetin isomer	3.2	3.2	2.9
Quercetin	18.2	12.0	14.8
Isoquercetin	3.8	1.2	2.3
Rutin	2.8	4.1	2.9
Quercitrin	3.4	1.3	2.7
Quinic acid	1.0	3.4	3.7
Raffinose	9.4	7.1	4.5
SAG	4.6	3.6	5.0
SAG isomer	9.5	6.5	6.8
Sedoheptulose-7P	13.4	8.4	6.7
Serine	4.4	5.2	2.2
Shikimic acid	5.3	5.0	4.1
Sinapic acid	10.1	16.1	17.9
Sorbitol	3.6	4.1	6.5
Stachyose	11.9	8.8	6.1
Succinic acid	7.2	5.4	7.0
Sucrose	1.6	1.8	2.2
Syringin	11.0	7.0	5.1
Tartaric acid	11.0	13.5	9.0
Threonate	6.2	4.8	5.3
Tricetin-G	3.0	2.5	3.5

Tricin		8.9	14.7	9.0
Tryptophan		5.7	7.0	6.2
Tyrosine		3.8	4.0	4.5
Tzeatin		12.3	11.3	10.0
TzeatinR		5.5	3.5	3.3
UDP-Glucose		5.0	4.7	5.0
Uric acid		18.2	12.4	13.4
Valine		0.8	7.8	3.2
Internal Standards	Alanine-d_3	5.8	5.6	3.8
	Apigenin-d_5	4.0	2.8	3.5
	N,N-Dimethyl-d_6-glycine	5.6	2.9	2.5
	Genistein-d_4	4.1	2.5	3.6
	Hippuric acid-d_5	2.6	2.4	2.3
	Methionine-d_3	3.9	5.9	3.7
	Phenylalanine-$^{13}C_6$	0.7	3.0	4.4
	Proline-d_3	2.4	9.3	11.9
	Salicylic acid-d_4	6.3	5.9	3.7

External QC	Precision between batches (RSD %)[a]
Alanine-d_3	9.45
Apigenin-d_5	20.37
N,N-Dimethyl-d_6-glycine	4.19
Genistein-d_4	21.02
Hippuric acid-d_5	11.20
Methionine-d_3	9.03
Phenylalanine-$^{13}C_6$	9.89
Proline-d_3	10.85
Salicylic acid-d_4	11.42

[a] Precision between batches were calculated for each analytical method (RP and HILIC) using internal standards

Supplementary Table S3. Results of biomarker selection by machine learning algorithms for classifying between resistant and susceptible mature trees.

Metabolite	Importance Score by RF[a]	Coefficient by SVM[b]	Estimate by Linear Regression[c]	FDR-adjusted *p*-value by Linear Regression[c]	Top 30%[d]
**Resistance Biomarkers**					
Syringin	9.216	0.192	1.473	0.000	Top 30% in Both
Kaempferol-3Rh	7.240	0.249	1.336	0.000	Top 30% in Both
Galactono-1,4-lactone	4.465	0.096	1.070	0.000	Top 30% in Both
Proline	6.236	0.137	0.918	0.000	Top 30% in Both
Glucose-6P isomer	4.127	0.108	0.619	0.001	Top 30% in Both
GABA	3.084	0.124	0.557	0.015	Top 30% in Both
Sedoheptulose-7P	2.051	0.139	0.761	0.000	Top 30% in Either
Chrysoeriol isomer	1.855	0.186	0.616	0.003	Top 30% in Either
Quercetin	0.929	0.105	0.466	0.017	Top 30% in Either
Genistein-7G	1.430	0.118	0.367	0.017	Top 30% in Either
Quinic acid	6.017	0.053	1.191	0.000	Top 30% in Either
Glycerate isomer	3.301	0.055	0.764	0.001	Top 30% in Either
Threonate	2.954	0.053	0.766	0.001	Top 30% in Either
Caffeic acid	3.263	0.045	0.379	0.004	Top 30% in Either
5-CQA	3.530	0.055	0.410	0.004	Top 30% in Either
Glucose	1.310	0.021	0.532	0.000	NR
Tyrosine	1.554	0.049	0.825	0.000	NR
Arabitol	2.536	0.087	0.752	0.000	NR
Galactose	1.113	0.030	0.561	0.001	NR
EGCG	1.560	0.027	-0.593	0.001	NR
GDP-Mannose	2.714	0.067	0.719	0.003	NR
Ferulic acid	1.606	0.051	0.357	0.005	NR
Dihydroquercetin	1.703	0.079	0.316	0.007	NR

Naringenin	2.061	0.055	0.406	0.021	NR
ATP Isomer	0.955	0.043	0.456	0.033	NR
Tryptophan	1.581	0.056	0.447	0.033	NR
ADP	2.752	0.063	0.387	NS	NR
Sucrose	0.490	0.027	0.283	NS	NR
Valine	1.492	0.040	0.274	NS	NR
Serine	1.901	0.097	0.164	NS	NR
Adenosine	0.812	0.066	0.163	NS	NR
Succinic acid	1.209	0.049	0.133	NS	NR
Astragalin	3.345	0.071	-0.049	NS	NR
Trifolin	0.810	0.086	-0.079	NS	NR
Phosphoglycerate	0.962	0.013	-0.118	NS	NR
Methyl-2-oxobutyric acid	1.680	0.233	-0.151	NS	NR
**Susceptibility Biomarkers**					
Apigenin-7G isomer	7.925	-0.218	-1.410	0.000	Top 30% in Both
Ascorbic acid	6.013	-0.160	-0.997	0.000	Top 30% in Both
Calycosin isomer	6.378	-0.101	-1.267	0.000	Top 30% in Both
Gallocatechin	3.560	-0.146	-0.850	0.000	Top 30% in Both
Calycosin	3.076	-0.254	-0.680	0.004	Top 30% in Both
Rutin	5.435	-0.088	-1.229	0.000	Top 30% in Either
Aspartate	3.545	-0.048	-0.923	0.000	Top 30% in Either
β-Glucogallin isomer	2.286	-0.167	-0.827	0.001	Top 30% in Either
Pinocembrin	2.927	-0.156	-0.366	0.001	Top 30% in Either
SAG	0.830	-0.131	-0.477	0.026	Top 30% in Either
myo-Inositol	1.691	-0.109	-0.359	0.045	Top 30% in Either
Glutamine	1.785	-0.057	-0.678	0.006	NR
GlcNAc	1.338	-0.025	-0.605	0.007	NR
SAG isomer	2.634	-0.082	-0.654	0.007	NR
Nictoflorin	2.518	-0.052	-0.575	0.010	NR
5-Aminopentanoate	1.728	-0.043	-0.548	0.017	NR

Leucine	0.910	-0.045	-0.552	0.024	NR
Ornithine	1.508	-0.092	-0.470	0.036	NR
Sorbitol	2.108	-0.090	-0.451	NS	NR
Arginine	1.382	-0.026	-0.443	NS	NR
Feruloylquinic acid	5.729	-0.183	-0.326	NS	NR
Citrulline	0.988	-0.018	-0.217	NS	NR
ATP	1.826	-0.042	-0.203	NS	NR
Asparagine	0.891	-0.026	-0.192	NS	NR
Stachyose	1.178	-0.081	-0.146	NS	NR
Deoxyadenosine	1.311	-0.091	-0.096	NS	NR
Chrysin	1.962	-0.051	-0.073	NS	NR
Biochanin A	4.083	-0.072	-0.004	NS	NR
Phenylalanine	0.932	-0.019	0.021	NS	NR
Alanine	0.715	-0.032	0.045	NS	NR
Raffinose	0.890	-0.040	0.045	NS	NR
Prunetin isomer	4.329	-0.077	0.162	NS	NR
Apigenin	2.461	-0.005	0.215	NS	NR
Guanosine	0.509	-0.004	0.229	NS	NR
Adenine	-0.255	-0.021	0.266	NS	NR
Uric acid	1.292	-0.016	0.301	NS	NR

[a] Importance score derived from random forest (RF) classification. The score reflects the decrease in model accuracy when the specific metabolite is excluded; higher values indicate greater importance for classification.
[b] Coefficients estimated by support vector machine (SVM) for classifying resistance vs. susceptible groups. Positive coefficients indicate association with the resistant group; negative coefficients indicate association with the susceptible group.
[c] Estimates from linear models comparing metabolite abundance between resistant and susceptible groups. Positive values indicate higher abundance in the resistant group; negative values indicate higher abundance in the susceptible group. P-values were adjusted using the false discovery rate (FDR) method. NS, not significant (FDR-adjusted $p \geq 0.05$).
[d] Top 30% of ranked metabolites based on importance in either or both classification methods. NR, not ranked in the top 30% by either method.

Supplementary Table S4. Results of biomarker selection by machine learning algorithms for classifying between resistant vs susceptible-like and resistant-like vs susceptible groups of mature trees in pecan leaflets.

Metabolite	Importance Score by RF[a]	Coefficient by SVM[b]	Estimate by Linear Regression[c]	FDR-adjusted *p*-value by Linear Regression[c]	Top 50%[d]
**Resistance Biomarkers: Resistant vs Susceptible-like**					
GDP-Mannose	3.424	0.064	1.041	0.000	Top 50% in Both
Glucose	1.550	0.056	0.701	0.000	Top 50% in Either
Serine	1.995	0.051	0.919	0.000	Top 50% in Either
Tyrosine	0.684	0.051	1.018	0.000	Top 50% in Either
Sedoheptulose-7P	3.509	0.047	0.771	0.011	Top 50% in Either
Sucrose	2.998	0.038	0.780	0.012	Top 50% in Either
Proline*	2.137	0.042	0.726	0.013	Top 50% in Either
Glycerate isomer*	3.303	0.037	0.701	0.028	Top 50% in Either
5-CQA*	2.801	0.048	0.456	0.028	Top 50% in Either
Galactose	1.140	0.045	0.709	0.001	NR
Galactono-1,4-lactone	1.331	0.037	0.618	0.014	NR
Trifolin	0.644	0.099	0.358	NS	Top 50% in Either
Quercetin*	0.101	0.067	0.500	NS	Top 50% in Either
Kaempferol-3Rh	2.685	0.061	0.406	NS	Top 50% in Both
Caffeic acid	-0.148	0.036	0.091	NS	NR
Valine	1.380	0.033	0.267	NS	NR
Dihydroquercetin	0.431	0.028	0.065	NS	NR
Threonate	-0.078	0.001	0.303	NS	NR
**Result of Resistant vs Susceptible-like: Related to Susceptibility-like**					
Chrysin	3.714	-0.156	-1.817	0.000	Top 50% in Both
Pinocembrin	3.256	-0.147	-1.690	0.000	Top 50% in Both
SAG	5.186	-0.116	-1.375	0.000	Top 50% in Both
Apigenin	2.337	-0.071	-1.269	0.000	Top 50% in Both

Calycosin	5.217	-0.185	-1.184	0.000	Top 50% in Both
Apigenin-7G isomer	4.495	-0.136	-1.047	0.000	Top 50% in Both
Feruloylquinic acid	4.122	-0.098	-1.034	0.001	Top 50% in Both
GlcNAc	1.336	-0.061	-0.782	0.005	Top 50% in Either
Calycosin isomer	1.872	-0.026	-0.233	0.012	NR
Sorbitol	0.278	-0.035	-0.438	NS	NR
ATP	-0.283	-0.037	-0.064	NS	NR
myo-Inositol	1.408	-0.051	-0.178	NS	NR
Nictoflorin	0.901	-0.111	-0.515	NS	Top 50% in Either
Gallocatechin	3.221	-0.118	-0.412	NS	Top 50% in Both
**Result of Resistant-like vs Susceptible: Related to Resistance-like**					
Syringin	2.916	0.112	0.844	0.000	Top 50% in Both
Genistein-7G	2.041	0.070	0.896	0.000	Top 50% in Both
Quinic acid	2.589	0.068	1.003	0.000	Top 50% in Both
Ferulic acid	1.712	0.063	0.623	0.000	Top 50% in Both
Glucose-6P isomer	1.786	0.067	0.737	0.005	Top 50% in Both
Arabitol	1.265	0.056	0.757	0.001	Top 50% in Either
Dihydroquercetin	2.064	0.038	0.878	0.011	Top 50% in Either
Tryptophan	0.921	0.084	0.457	0.036	Top 50% in Either
Kaempferol 3-*O*-α-L-rhamnoside	1.192	0.016	0.492	0.000	NR
Galactose	0.948	0.046	0.657	0.002	NR
Glucose	0.641	0.045	0.676	0.002	NR
Succinic acid	-0.253	0.059	0.339	NS	Top 50% in Either
Sedoheptulose-7P	0.299	0.026	0.239	NS	NR
5-CQA	1.718	0.010	0.023	NS	Top 50% in Either
**Susceptibility Biomarkers: Resistant-like vs Susceptible**					
Rutin*	4.389	-0.096	-1.356	0.000	Top 50% in Both
5-Aminopentanoate	4.422	-0.088	-1.360	0.000	Top 50% in Both
Gallocatechin*	4.054	-0.087	-1.174	0.000	Top 50% in Both

Nictoflorin	3.025	-0.114	-1.054	0.000	Top 50% in Both
Leucine	3.308	-0.086	-1.186	0.001	Top 50% in Both
Calycosin*	2.741	-0.111	-0.909	0.003	Top 50% in Both
Phenylalanine	2.597	-0.054	-0.634	0.025	Top 50% in Both
Pinocembrin*	2.324	-0.063	-0.360	0.036	Top 50% in Both
Glutamine	2.681	-0.050	-1.242	0.000	Top 50% in Either
Calycosin isomer*	3.330	-0.050	-1.090	0.003	Top 50% in Either
GlcNAc	2.621	-0.043	-0.886	0.004	Top 50% in Either
Asparagine	0.817	-0.061	-0.821	0.005	Top 50% in Either
Aspartate	1.201	-0.047	-1.184	0.000	NR
Ornithine	0.845	-0.009	-0.765	0.021	NR
Chrysin	0.886	-0.017	0.041	NS	NR
Sorbitol	0.160	-0.017	-0.188	NS	NR
SAG	0.914	-0.022	-0.520	NS	NR
Arginine	0.887	-0.026	-0.649	NS	NR
Feruloylquinic acid	3.430	-0.040	0.073	NS	Top 50% in Either
Alanine	1.148	-0.045	-0.516	NS	NR
Deoxyadenosine	0.849	-0.048	-0.326	NS	NR
Adenine	0.348	-0.048	-0.166	NS	NR
β-Glucogallin isomer	1.501	-0.055	-0.368	NS	Top 50% in Either
Prunetin isomer	1.218	-0.055	0.125	NS	Top 50% in Either

[a] Importance score derived from random forest (RF) classification. The score reflects the decrease in model accuracy when the specific metabolite is excluded; higher values indicate greater importance for classification.
[b] Coefficients estimated by support vector machine (SVM) for classifying between resistant and susceptible groups. Positive coefficients indicate association with the resistant group; negative coefficients indicate association with the susceptible group.
[c] Estimates from linear models comparing metabolite abundance between resistant and susceptible groups. Positive values indicate higher abundance in the resistant group; negative values indicate higher abundance in the susceptible group. P-values were adjusted using the false discovery rate (FDR) method. NS, not significant (FDR-adjusted $p \geq 0.05$).
[d] Top 50% of ranked metabolites based on importance in either or both classification methods. NR, not ranked in the top 50% by either method.
* Biomarkers overlapped with the original comparison of resistant vs. susceptible (Supplemental table S3).

Supplementary Table S5. Cohen's *d* values summarizing standardized differences in log_2 transformed metabolite abundances across scab resistance groups. Effect sizes for susceptibility biomarkers were calculated relative to the Resistant group, whereas resistance biomarkers were calculated relative to the Susceptible group; therefore, the reference group values are zero by definition. Positive values indicate higher abundance relative to the respective reference group, and negative values indicate lower abundance. Values with |d| > 0.5 are shown in bold to denote medium to large effect sizes.

Biomarkers	Susceptible	Susceptible-like	Resistant-like	Resistant
Resistance Biomarkers				
Syringin	0	**0.749**	**1.437**	**1.865**
Kaempferol-3Rh	0	**0.937**	**1.173**	**1.783**
Quinic acid	0	**0.581**	**1.287**	**1.421**
Galactono-1,4-lactone	0	0.484	0.242	**1.357**
Proline	0	0.422	**0.551**	**1.173**
Glucose	0	-0.300	**0.958**	**0.952**
Tyrosine	0	-0.331	0.222	**0.938**
Sedoheptulose-7P	0	-0.013	0.330	**0.889**
Glycerate isomer	0	0.076	0.093	**0.775**
Threonate	0	**0.524**	-0.107	**0.759**
Glucose-6P isomer	0	0.395	**0.844**	**0.757**
5-CQA	0	-0.192	0.300	**0.730**
Chrysoeriol isomer	0	**1.446**	0.329	**0.702**
Caffeic acid	0	**0.594**	**0.654**	**0.698**
GDP-Mannose	0	-0.331	0.227	**0.696**
GABA	0	**0.683**	0.223	**0.693**
Genistein-7G	0	**1.960**	**1.234**	**0.619**
Quercetin	0	-0.050	0.456	**0.551**
Sucrose	0	**-0.561**	-0.117	0.282
Serine	0	**-0.728**	-0.465	0.215
Susceptibility Biomarkers				
Apigenin-7G isomer	**2.040**	**1.417**	**1.615**	0
Calycosin isomer	**1.743**	**0.792**	0.476	0
Rutin	**1.684**	0.141	-0.321	0
Ascorbic acid	**1.517**	**1.171**	**0.993**	0
Gallocatechin	**1.026**	0.333	**-0.580**	0
Aspartate	**0.919**	0.385	-0.317	0
Glutamine	**0.779**	-0.296	**-0.792**	0

Calycosin	**0.764**	**1.581**	-0.222	0
Pinocembrin	**0.748**	**1.617**	0.012	0
β-Glucogallin isomer	**0.723**	-0.265	**0.749**	0
GlcNAc	**0.671**	**0.784**	**-0.511**	0
Nictoflorin	**0.581**	0.434	**-0.780**	0
5-Aminopentanoate	**0.548**	**-0.613**	**-1.027**	0
SAG	**0.505**	**1.661**	-0.049	0
Leucine	0.460	**-0.662**	**-1.172**	0
myo-Inositol	0.456	0.234	0.152	0
Asparagine	0.198	**-1.186**	**-0.808**	0
Phenylalanine	-0.101	0.456	**-0.973**	0

Supplementary Figure S1. Workflow of biomarker identification using machine learning.

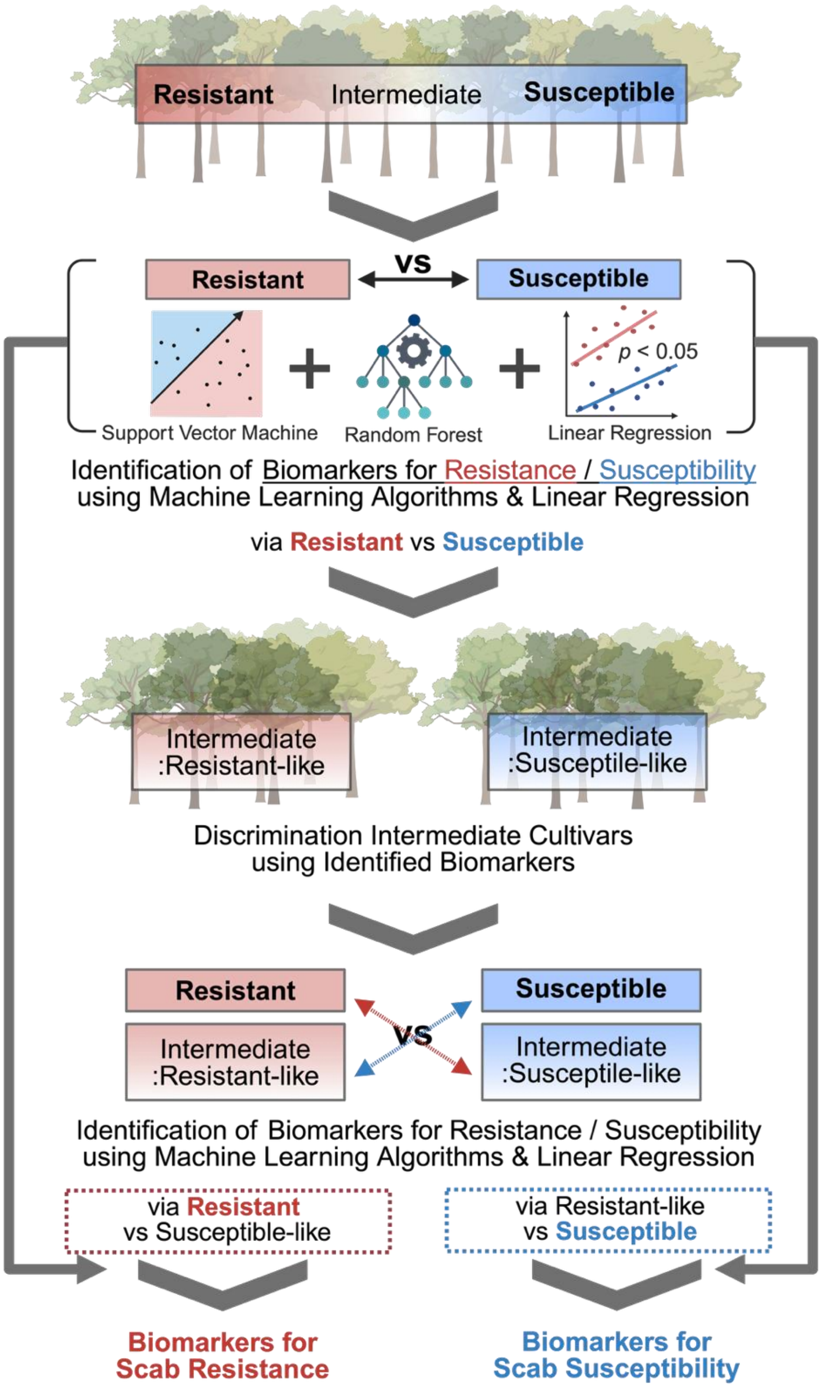

Supplementary Figure S2. Permutation test results of classification between resistant vs susceptible using support vector machine and random forest.

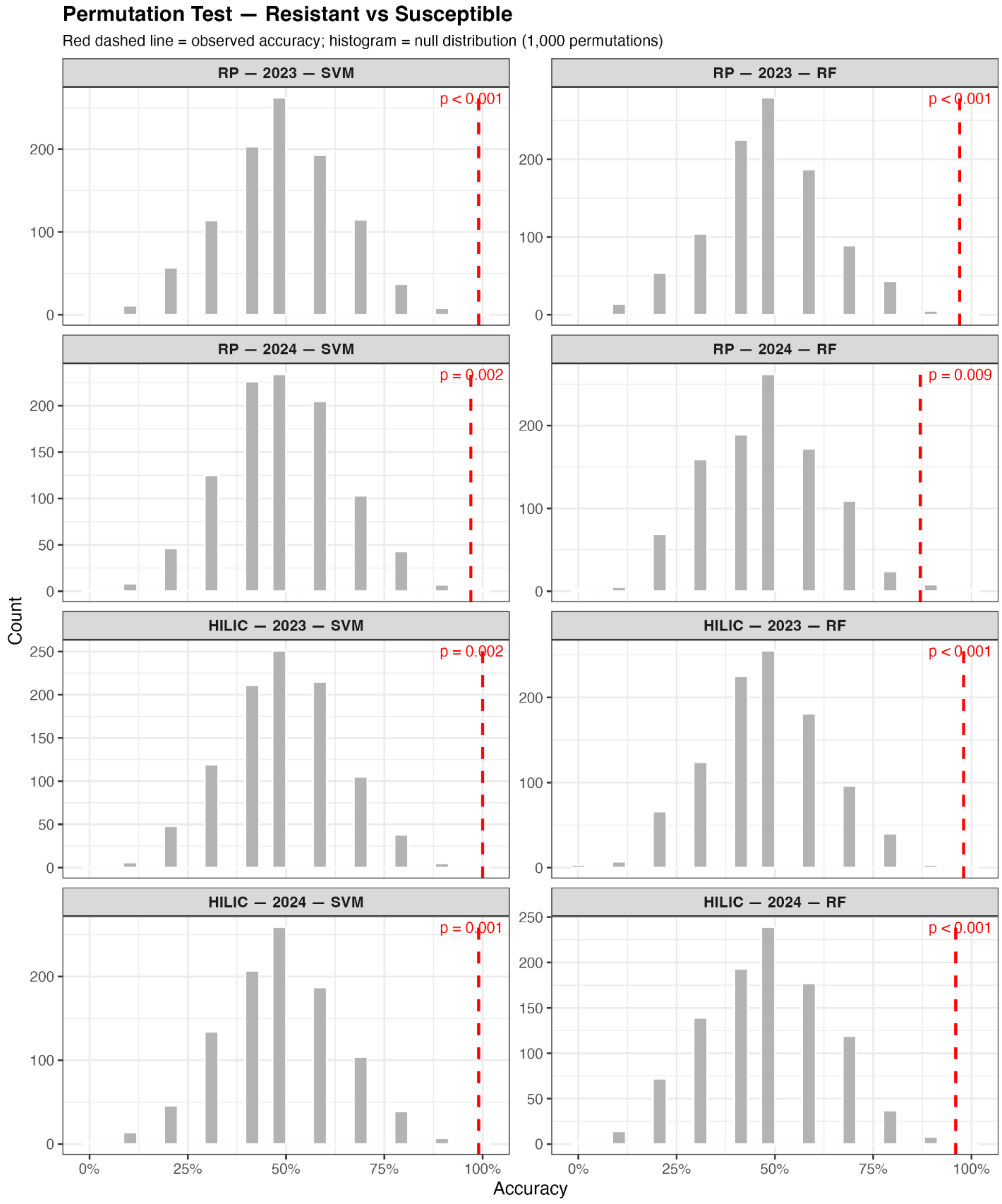

Supplement: Supplementary file 1 [file jf5c16064_si_001.pdf]
